# Linear and Nonlinear Optical Properties of Azobenzene Derivatives Modified with an (Amino)naphthalene Moiety

**DOI:** 10.1021/acs.jpcb.2c03078

**Published:** 2022-08-09

**Authors:** Marta Dudek, Anna Kaczmarek-Kędziera, Radosław Deska, Jakub Trojnar, Patryk Jasik, Piotr Młynarz, Marek Samoć, Katarzyna Matczyszyn

**Affiliations:** † Institute of Advanced Materials, Faculty of Chemistry, Wrocław University of Science and Technology, Wyb. Wyspiańskiego 27, 50-370 Wrocław, Poland; ‡ Faculty of Chemistry, Nicolaus Copernicus University in Toruń, Gagarina 7, 87-100 Toruń, Poland; § Faculty of Applied Physics and Mathematics and BioTechMed Center, Gdańsk University of Technology, Gabriela Narutowicza 11/12, 80-233 Gdańsk, Poland; ∥ Department of Biochemistry, Molecular Biology and Biotechnology, Faculty of Chemistry, Wrocław University of Science and Technology, Wyb. Wyspiańskiego 27, 50-370 Wrocław, Poland

## Abstract

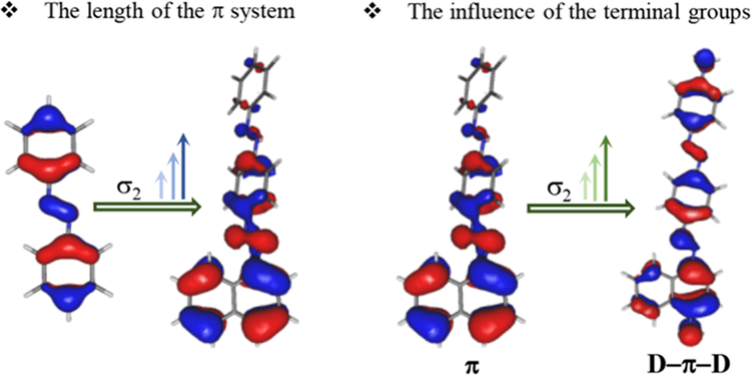

The design of two-photon absorbing azobenzene (AB) derivatives has received much attention; however, the two-photon absorption (2PA) properties of bis-conjugated azobenzene systems are relatively less explored. Here, we present the synthesis of six azobenzene derivatives and three bis-azobenzenes substituted (or not) at *para* position(s) with one or two amino group(s). Their linear and nonlinear absorption properties are studied experimentally and theoretically. The switching behavior and thermal stability of the *Z*-isomer are studied for unsubstituted mono- (**1a**, **2a**) and bis-azobenzene (**3a**) compounds, showing that when the length of the π system increases, the half-life of the *Z*-isomer decreases. Moreover, along with the increase of π-conjugation, the photochromic characteristics are impaired and the photostationary state (PSS) related to *E*–*Z* photoisomerization is composed of 89% of the *Z*-isomer for **2a** and 26% of the *Z*-isomer for **3a**. Importantly, the 2PA cross-section increases almost five-fold on extending the π-conjugation (**2a** vs **3a**) and by about one order of magnitude when comparing two systems: the unsubstituted π-electron one (**2a**, **3a**) with D-π-D (**2c**, **3c**). This work clarifies the contribution of π-conjugation and substituent effects to the linear and nonlinear optical properties of mono- and bis-azobenzene compounds based on the experimental and theoretical approaches.

## Introduction

The interest in development of molecular switches^1−6^ arises from the fact that imparting external sensitivity to molecular systems is an efficient way to control on-demand their structure, properties, and functions and hence offers multiple applications in materials^7−11^ and biological sciences.^12−15^ Among various external triggers, light is the most desired kind of stimulus, because spatiotemporal resolution, excitation tunability, and biocompatibility are achieved through remote control.^12,16,17^ A particularly important group of light-sensitive compounds in this context are azobenzenes (ABs).^18^ The physicochemical and structural changes accompanying their *E*–*Z* photoisomerization, i.e., the photochromic effect, have been widely exploited to provide the strategy for material modification by means of light stimulation.^19,20^ Up to now, azo compounds have been used to modulate the properties of supramolecular systems,^21−23^ biomolecules (DNA, protein),^24,25^ ion channels and receptors,^26,27^ polymers,^28^ and liquid crystals,^29,30^ in solution as well as on surfaces and bulk materials, transferring effects from the molecular level to the macroscopic scale.^31^ In general, the *E*–*Z* isomerization is induced by UV irradiation and the return to the initial state can be achieved either by visible light or thermally.^32,33^ However, especially in biology, due to limitations concerning one-photon excitation such as light penetration depth or toxicity of UV light,^34^ the two-photon (2P) excitation of the photochromic molecules may appear useful.^27,35^ The longer excitation wavelengths provide deeper and safer tissue penetration in comparison to that of the conventional linear (one-photon) absorption species. So far, a multitude of 2P-absorbing compounds have been synthesized,^36^ including AB derivatives^10^ and their nonlinear optical (NLO) absorption properties have been exploited in both biological^27^ and materials sciences.^37,38^ However, exploiting 2PA generally requires high light intensities, such as those available from a focused short-pulse laser beam, to reach the desired therapeutic/diagnostic action. To alleviate the need for high pulse powers, a continuous search for efficient 2PA materials^10,39^ exhibiting high 2PA cross-section values is ongoing. The strategies developed for new NLO dyes^36^ include the elongation of the coplanar π-electron scaffold and introduction of electron-donating groups (EDG) leading to the D−π–D architecture. In the case of ABs, the conjunction of azo- and bisazo-chromophores with the expanded π-electron delocalized skeleton with the addition of terminal EDGs may yield potent 2PA agents with relevant photochromic characteristics.

**Figure 1:**
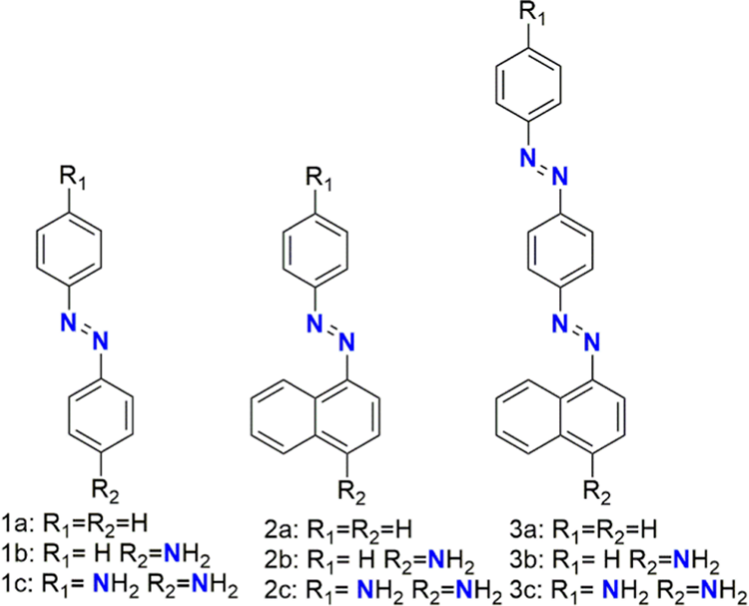
Molecular structures of the investigated compounds.

We report on the design, synthesis, and linear and nonlinear optical characterization of nine AB derivatives, six of them (**1a**–**2c**) possessing one azo group and three (**3a**–**c**) possessing two azo groups (Figure 1). Our studies of the contributions of π-conjugation and substituent effect to the properties of AB molecules indicate that substitution of AB with a donating group (here amine) impacts significantly both the absorption spectra (red-shifted compared to unsubstituted AB) and the thermal half-life of the *Z*-isomer (significant decrease).^32,33^ In view of this, we investigated the composition of the photostationary state (PSS) and thermal half-life of the *Z*-isomer for **2a** and **3a** using **1a** (azobenzene) as a reference compound and we show that the photochromic properties can be easily tuned by the modification of the length of the π-system. The two-photon absorption properties of all compounds were studied using the *Z*-scan method. It appears that the molecular design strategy, based on well-known ways to boost the third-order NLO properties like the extension of π-conjugated chains with (mostly) enforced coplanarity and attaching donor group(s) at the end(s) of the molecule^36^ enabled us to significantly enhance the 2PA properties and to draw some conclusions concerning the structure–2PA property relationship. Additionally, to clarify the origin of the experimentally observed results, quantum-chemical calculations were performed.

## Experimental Section

### Synthetic Methodology

**Scheme 1:**
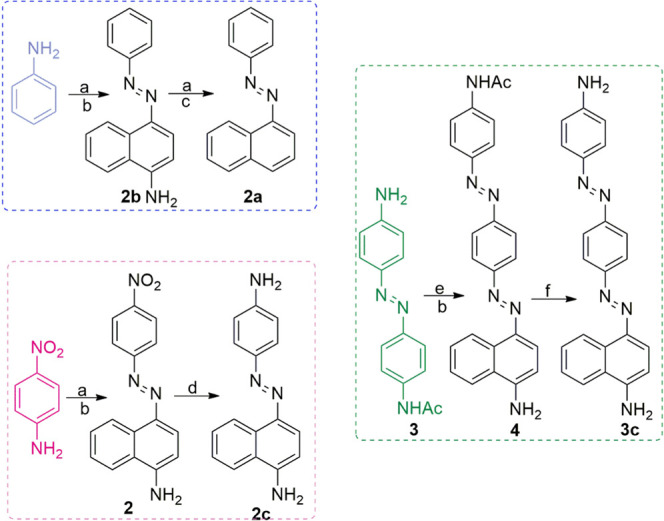
Syntheses of **2a**-**c** and **3c**. Experimental conditions: (a) NaNO_2_, HCl, ∼0 °C; (b) 1-naphthylamine; (c) H_3_PO_2_, (d) Na_2_S, THF/H_2_O 3/1, Δ; (e) NaNO_2_, HBF_4_, ∼0 °C, and (f) HCl/MeOH, Δ

Some of the studied compounds are commercially available, however, except for **1b**, the azo molecules were synthesized by us, implementing a new synthetic strategy (Scheme 1). The syntheses of **1a**,^40^**1c**,^25^ and **2b**(41) have already been reported, so here only the synthetic route used to obtain other AB derivatives: **2a**, **2c** (modified synthesis conditions),^42^**3a**, **3b**, and **3c** will be described briefly. Molecules **2a** and **2c** were synthesized in two steps, starting from the commercially available aniline or 4-nitroaniline, which were first subjected to a classical diazonium salt coupling^43^ for obtaining **2b** or **2** (Scheme 1). To get compound **2a**, we transformed the amino group (**2b**) into diazonium chloride and then we used hypophosphorous acid as a mild reducing agent to finally get **2a**. **2** was chemically reduced to its amine analogue, **2c**.^42^ Compounds **3a** and **3b** were synthesized starting from 4-aminoazobenzene, employing the same synthetic route as for **2a** and **2b**. Compound **3c** was obtained in four steps. First, two steps including Mills reaction and reduction of the vitro to amino group were utilized to get **3** (see the Supporting Information (SI) for details pp. S2–S16). Then the diazonium salt was prepared from **3** and directly coupled with 1-naphthylamine to give **4**, and after deprotection of the amine group, compound **3c** was obtained.

### Sample Irradiation

The photoinduced isomerization reactions of **1a**, **2a**, and **3a** were performed using a UV spot-light source (Hamamatsu Photonics K.K., model: L9588-04) equipped with filters operating at 313, 365, 405, 436, and >485 nm (Figure S14).

### Photochemical Behavior

UV–Vis absorption experiments were carried out on a JASCO V-730 spectrophotometer equipped with the JascoPeltier type temperature controller (CDF-426S/15) at 25 °C. All optical measurements were performed in quartz cuvettes with path lengths of 10 mm. The 30 μM solutions of the investigated molecules in dimethyl sulfoxide (DMSO) and dichloromethane (DCM) were prepared and the UV–Vis absorption spectra were recorded before and after irradiation with light of appropriate wavelength.

### Thermal Stability of the *Z* Isomers

To analyze the thermal relaxation process of the *Z*-isomer, the absorbance changes were measured at different temperatures as a function of time. First, the 30 μM solutions of **1a**, **2a**, and **3a** in acetonitrile were irradiated with light: **1a** – 313 nm, **2a** – 365 nm, and **3a** – 405 nm for 15 min to reach the PSS. Then, absorbance readings were taken between 220–600 nm with 60 s or 90 s intervals at 50, 55, 60, and 65 °C for **1a** and **2a** and 20, 30, 35, and 40 °C for **3a** (see the Supporting Information for details pp. S17–S20).

### Composition of the PSS

The 60 μM hexane solution of **1a**, **2a**, and **3a** were irradiated with light of appropriate wavelength for 15 min before each measurement: **1a** – 313 and 436 nm, **2a** – 365 and 436 nm, and **3a** – 405 and 313 nm. Then the sample was immediately injected into the analytical high-performance liquid chromatography (HPLC) column (normal phase, UV–Vis detection). The experiment was performed under isocratic conditions (90/10 hexane/isopropanol) using a CHIRALPAK IB column with a flow rate of 1.0 mL min^–1^. The PSS composition was determined by the integration of the UV signal at wavelengths of the isosbestic points (where the molar absorption coefficients for photoisomers are the same).

### *Z*-scan Studies

The 2PA cross-sections of AB derivatives in solution were studied for samples in their thermally relaxed state. All samples were dissolved in DMSO at the concentrations given in Table 2, and placed in 1 mm glass cuvettes to perform *Z*-scan measurements. Given that the one-photon absorption (1PA) of the investigated compounds takes place in the UV–Vis region and the samples are transparent in the near-infrared region, the NLO measurements were carried out in the one-photon transparency region. The details about the *Z*-scan experimental setup can be found in the literature.^10,36,39,44^ In this technique, the cuvette containing the sample solution is moved along the *z*-axis of a focused beam (*z* = 0 corresponding to the focal plane), and the transmittance of a nonlinear medium is measured in two ways: (i) open-aperture (OA) *Z*-scan traces, where the total transmitted power is recorded and (ii) closed-aperture (CA) traces, where an aperture is placed in the far-field and the transmittance through this aperture is recorded. In our experiments, we always recorded both CA and OA traces simultaneously, through the use of a beam splitter in the path of the beam after the sample, with two separate detectors. The nonlinear optical experiments were performed by employing laser pulses from an optical parametric amplifier (Light Conversion TOPAS Prime) pumped by 70-fs pulses at 800 nm delivered by a Coherent Astrella Ti: sapphire regenerative amplifier system with a repetition rate of 1 kHz. The output beam was selected with a polarization separator and attenuated using neutral density filters. The pulse energy and the focusing were adjusted to keep the light intensities in the range of 60–90 GW cm^–2^. Results obtained for the solutions of the investigated compounds in DMSO were calibrated against *Z*-scan measurements on a fused silica plate (for which the values of the nonlinear refractive index (*n*_2_) as a function of the wavelength are well established) and compared with the measurements on an identical glass cell filled with the pure solvent (DMSO). The obtained data were analyzed using a custom fitting program that utilized equations derived by Sheik-Bahae et al.^44^ Briefly, the fitting procedure involves the determination of the nonlinear phase shifts, and then the nonlinear refractive index and the nonlinear absorption coefficient α_2_ of the solution are calculated. The CA traces can be analyzed in our software directly for obtaining both n_2_ and α_2_ or, alternatively, the value of *n*_2_ is determined from a trace that is obtained by dividing the CA trace by the corresponding OA trace and the value of α_2_ is determined by fitting the OA trace. The nonlinear (two-photon) absorption coefficient is then used to calculate the two-photon absorption cross-section using equation^45^
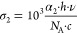
1where *N*_A_ is the Avogadro constant, *c* is the concentration of the compound in solution (in mol/dm^3^), *h* is the Planck constant, and ν is the frequency of the incident laser beam.

## Theoretical Calculations

The full geometry optimization of the analyzed molecules has been performed within the ωB97X-D/def2-TZVP approach in DMSO solvent described using the polarizable continuum model. The character of the stationary points on the potential energy surface has been confirmed by harmonic frequency analysis. Vertical excitation energy for one-photon absorption has been estimated with the ωB97X-D and CAM-B3LYP functionals with the def2-TZVP basis set, with the corrected linear response solvation. Because of the large number of molecular orbitals involved in the transitions, particularly for multichromophoric systems **3a**–**3c**, the natural transition orbitals have been drawn to visualize the character of the observed excitations.^46^ The two-photon absorption of the linearly polarized photons of the same energy was analyzed by the CAM-B3LYP/def2-TZVP approach in DMSO as well. The choice of the CAM-B3LYP functional was rationalized by its systematic behavior with respect to the estimated 2PA strengths for organic molecules.^47−49^ The cross-section for the two-photon absorption, given in Goeppert-Mayer units,^48,49^ is defined as
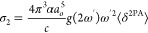
2where α is the fine structure constant, *a*_0_ corresponds to the Bohr radius, c denotes the speed of light in a vacuum, ω′ stands for the photon angular frequency, and *g*(2ω′) is the line shape function, assumed here as a Gaussian profile with the arbitrary broadening factor equal to 0.1 eV. The rotationally averaged 2PA strength ⟨δ^2PA^⟩ in atomic units is given as
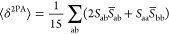
3with *S* being the second-order transition moments between the 0 and final states. The precise definition of these transition moments can be found elsewhere.^48,49^

The 2PA calculations have been performed using Dalton2015,^50,51^ and all of the remaining calculations have been carried out using Gaussian16.^51,52^ This choice of software is typical in similar cases.^47,48^

## Results and Discussion

**Figure 2:**
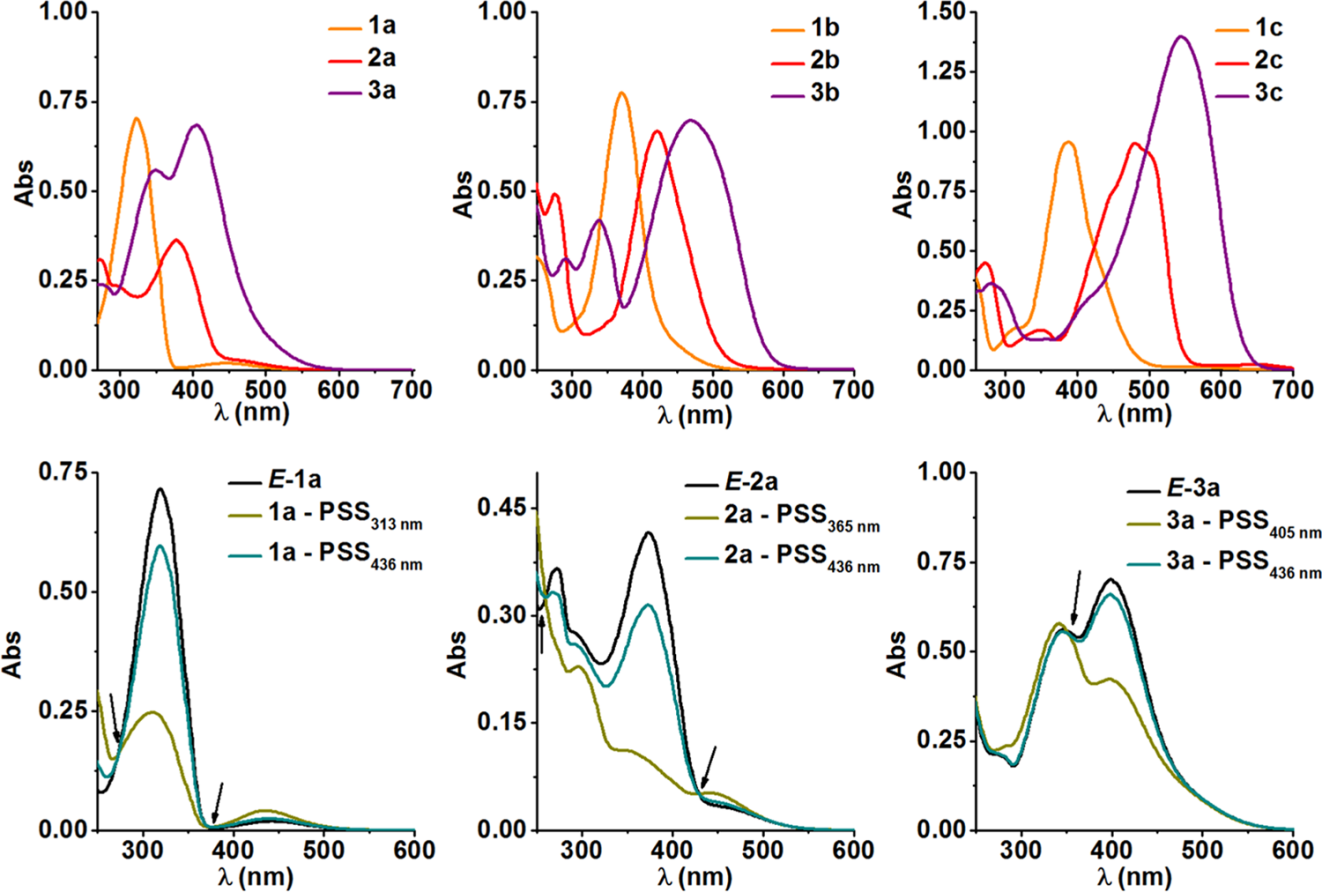
Absorption spectra of 30 μM DMSO solutions of the investigated compounds (upper panel) as well as their PSS mixtures (lower panel) for **1a**, **2a**, and **3a** in DCM, all at 25 °C. The bold arrows aim to show the appearance of isosbestic points.

The absorption spectra of all studied compounds were measured in DMSO and the spectra corresponding to PSS mixtures for **1a, 2a**, and **3a** were measured in DCM and are displayed in Figure 2. Their structure can be understood as arising from two types of transitions. The dominant absorption maxima, present at higher energies, can be assigned to π–π* S_0_ → S_2_ vertical transitions with molar absorptivity coefficients at the respective band maxima in the range (12–47)·10^3^ M^–1^ cm^–1^ (Table 1). The second band, generally observed for AB derivatives, corresponds to *n*–π* S_0_ → S_1_ transition and tends to be of much smaller oscillator strength due to symmetry considerations.^33^ Indeed, for **1a** and **2a** the second peak can be observed with maxima at 437 and 462 nm, respectively. For the rest of the studied compounds the bands corresponding to the *n*–π* transitions are masked by the π–π* bands that are strongly bathochromically shifted as a consequence of the extended π-conjugation along with the increase of electronic conjugation by attaching further phenyl rings, the absorption maxima (π–π* transition) are red-shifted,^53^ from 319 nm for **1a** to 400 nm for **3a**, from 371 nm for **1b** to 470 nm for **3b**, and from 389 nm for **1c** to 545 nm for **3c** (Figure 2). The same tendency can be observed if one takes into account the number of amino groups present in the molecules: 0 (**1**-**3a**), 1 (**1**-**3b**), or 2 (**1**-**3c**). This relatively strong electron-donating group (EDG) (σ_p_ = −0.66)^54^ pushes electrons onto the ring and hence increases the electron density in the ring and red-shifts the absorption maximum (Figures S26–S28).

**Table 1:** Experimentally (exp) found absorption maxima with the corresponding molar absorption coefficient and calculated (calc) geometrical parameters after optimization using the ωB97X-D/def2-TZVP/PCM(DMSO) level of theory.

cmpd		maxima of the absorption bands λ^exp^ (nm) [ε(10^3^ M^–1^cm^–1^)]	ring torsion^b^ (deg)	dihedral −C-N=N–C (deg)	λ_1PA_^calc^ (nm)	Δμ_gf_^e^ (D)	μ_gf_^f^ (au)
**1a**	***E***	323 [23.6]	0.00	–180.00	310	0.00	3.08
		446 [0.88]			454	0.00	0.00
	***Z***^a^	434 [1.3]	–31.1	6.44	433	–0.44	0.61
**1b**		370 [25.8]	–0.2	–179.99	349	6.40	3.49
**1c**		389 [31.8]	0.0	–179.99	365	0.00	3.81
**2a**	***E***	377 [12.1]	47.1	–179.78	328	4.12	2.64
	***Z***^a^	445 [1.3]	–30.4	3.71	437	–0.06	0.56
**2b**		421 [22.1]	–16.8	–179.39	378	4.32	3.46
**2c**		480 [31.6]	–15.3	–179.88	386	0.24	3.71
				441		1.04	
**3a**	***E***_**1**_***E***_**2**_	346 [18.6]	–45.5	179.58^c^	361	2.55	1.32
		405 [22.8]	–3.5	179.88^d^	479	2.83	4.24
	***E***_**1**_***Z***_**2**_			–179.89^c^	323	0.35	1.03
				6.71^d^			
	***Z***_**1**_***E***_**2**_			3.84^c^	338	2.71	2.99
				–179.85^d^			
	***Z***_**1**_***Z***_**2**_			3.98^c^	283	–0.83	1.12
				6.50^d^			
**3b**		338 [14.0]	–12.0	179.29^c^	417	1.25	4.80
		470 [23.2]	–0.9	179.94^d^	466	7.32	0.96
**3c**		545 [46.6]	–10.7	179.42^c^	424	0.78	5.25
			–0.8	179.88^d^	462	3.27	1.25

a Given for PSS_*Z*_, found experimentally.

b dihedral angle between the planes of phenyl rings.

c –C1-N12=N13-C14–.

d –C34-N33=N9-C4–, see Supporting Information for atom numbers, Figure S23.

e Δμ_gf_ - difference between ground and excited-state dipole moments.

f μ_gf_ - transition dipole moment.

**Figure 3:**
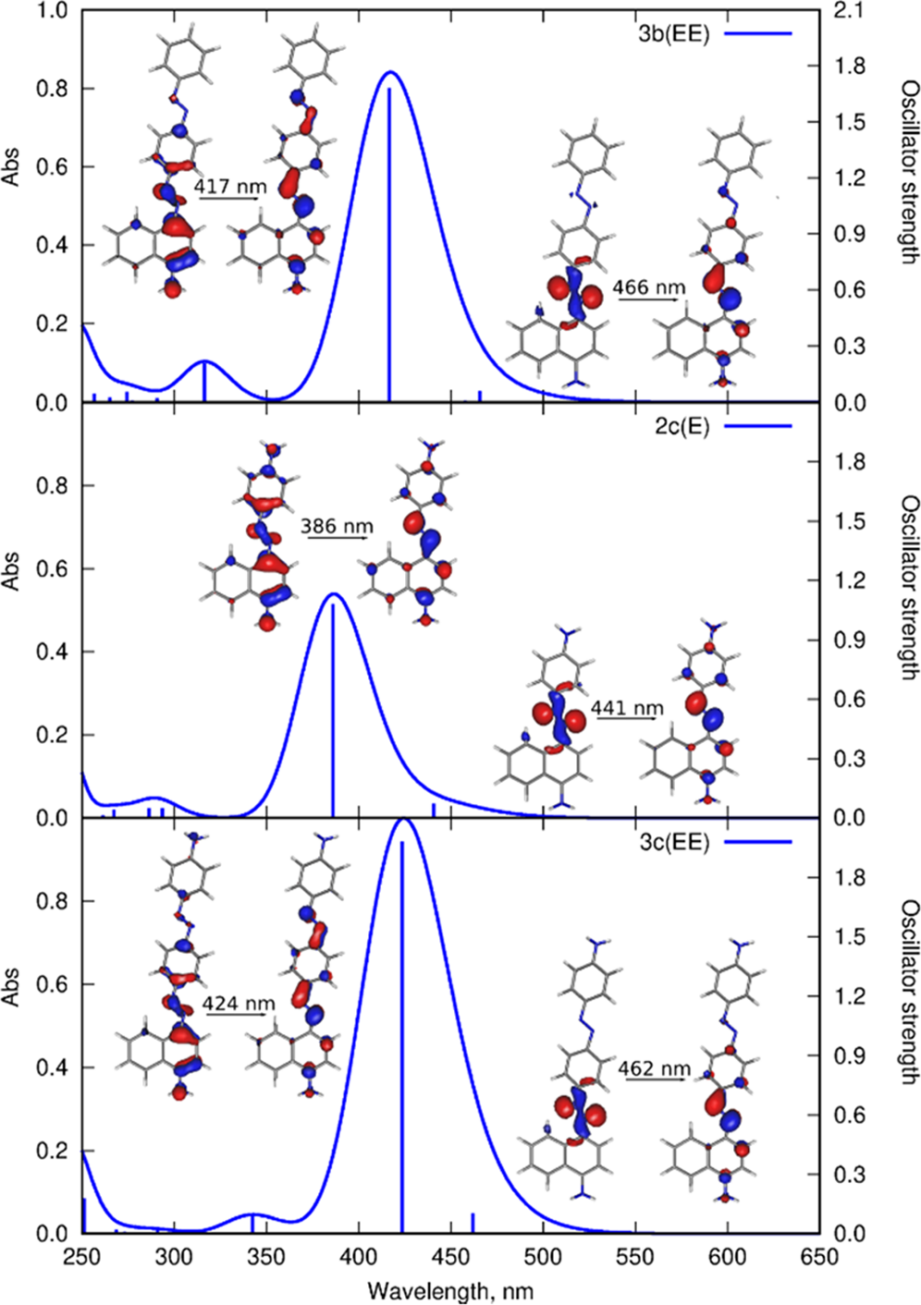
Vertical absorption spectrum estimated within the ωB97X-D/def2-TZVP/PCM(DMSO) approach for **3b**, **2c**, and **3c**: the sticks represent the predicted transitions and the envelope is computed by assigning Gaussian bands to all of the transitions. Natural transition orbitals involved in the most important transitions are presented in the insets.

The experimental findings detailed above are well supported by theory. Computationally derived absorption spectra are presented in Figures 3 and S26–S28. They reveal a significant influence of the π-electron scaffold extension and substituent effects on the energy for the first vertical electronic transition for a given compound (Figures 3, S29 and S30). Although a little underestimated by the calculations, a fair match is found between the computed and measured first transition energies, mostly blue-shifted as previously observed.^55,56^ While the simulated spectra for the *E* isomer of **1b**, **1c**, **3b**, and **3c** reproduce the apparent merging of π–π* and *n*–π* transitions (Figures 3 and S29), the presence of weak (*n*–π*) transitions for **2b**, **2c**, and **3a** was not experimentally observed (Figure 2). The calculated spectra for the *Z*-isomer of **1a** and **2a** (Figures S29 and S30) are in good agreement with those found experimentally. The natural transition orbital analysis of the *E* isomer enabled us to assign the π–π* character to the intense band and *n*–π* character to the weak transition (Figures 3, S29 and S30). The natural transition orbitals involved in the corresponding transitions are presented in the insets in Figure 3. It can be noticed that, due to the strong electron-donating character of the NH_2_ substituent, in the case of **1b** and **2b***E*-isomers, the HOMO orbital is mostly concentrated within the substituted phenyl ring, while in the case of unsubstituted **1a** or **2a** and doubly-substituted **1c** and **2c** molecules, both phenyl rings contribute equally, as can be expected. On the other hand, for the LUMO orbital shape, the substitution has only a minor influence, since the electron density is concentrated on the diazo bridge and undergoes only a tiny modification upon NH_2_ introduction in **1b** or **2b***E*-isomers.

Using DFT calculations at the ωB97X-D/def2-TZVP/PCM(DMSO) level of theory we performed the natural bond orbital (NBO) analysis for minimum energy structures and we found that the calculated charges at the nitrogen of the amino group(s) differ only subtly depending on the substitution: phenyl or naphthalene, as well as the distance between the amino groups (**2c**–**3c**) (Figure S24). However, a substantial modification of the charge distribution is observed on the diazo bridges, both upon extension of the delocalized π-electron skeleton and upon introduction of an electron-donating substituent. For instance, for the N12–N13 bridge (Figure S23), the NBO negative charge increases from −0.3280 to −0.3722 in the **1a**–**1c** series, from −0.3255 to −0.3891 for **2a**–**2c** series, and from −0.3264 to −0.3901 for **3a**(*EE*)–**3c**(*EE*) series (Figure S24). This clearly indicates the growing electron-donating strength of the substituents in the dyes. Furthermore, the second-order perturbation theory analysis of the corresponding Fock matrices in the NBO basis showed that the coupling between nitrogen and the aromatic ring increases with the growth of the π-electron delocalized molecular scaffold. It is clearly visible in the sequence of systems containing only one amino group, namely **1b**, **2b**, and **3b**, where the donor–acceptor interaction contributes to the overall stabilization respectively by 48.37, 53.48, and 57.83 kcal mol^–1^ (Table S3). This input arises mainly from the one-electron Hamiltonian integrals, with almost the same donor and acceptor orbital energies for all of the molecules. A similar monotonic tendency is also observed for the conjugation of the NH_2_-group at the naphthalene/phenyl ring in the systems bearing two amino substituents. The influence of the amino group(s) can also be verified by the ground state dipole moment vectors (Table S5). Substitution of **1a**, **2a**, and **3a** with amino group(s) obviously increases the dipole moments. Moreover, as the π-conjugation length is enhanced, significant growth of the dipole moment is also observed, namely in the sequence **1b** → **2b** → **3b**, the values of 3.95 D, 5.01 D, and 6.11 D are obtained, respectively (Table S5). The most important features affecting the transition energy as well as permanent and transition dipole moments involved in the 1- and 2PA are the torsion angle between the planes of aromatic rings and the dihedral angle (Table 1). The closer the value of the torsion angle is to 0° and that of the dihedral angle (−C–N=N–C−) to 180°, the more planar a molecule is and the larger the possible π-electron delocalization is. It can be seen (Table 1) that the dihedral angle for the *E* isomer of the investigated compounds is maintained at almost 180°. The most pronounced deviation from planarity for the *E* isomer is noticed in the case of the unsubstituted systems containing the naphthalene moiety (**2a** and **3a**), however, it does not seem to significantly affect the ground state (GS) dipole moments (Table S5).

Upon irradiation at the dominant absorption maxima: 313 nm (**1a**), 365 nm (**2a**), and 405 nm (**3a**) the intense band corresponding to the π–π* transition decreases, and the band attributed to the *n*–π* transition increases for **1a** and **2a** until the PSS (*E*-to-*Z* isomerization) is reached. Irradiation with blue (436 nm) or UV (313 nm) light induces *Z*–*E* isomerization and the intensity of the initial π–π* transition is restored (Figure 2). Importantly, the decrease in the π–π* transition band of **3a** is unusually small, indicative of a *Z*-poor PSS. Moreover, in contrast to **2a**, **3a** does not possess distinct isosbestic points indicating that as **3a** is a two *para*-connected AB motif, probably a high degree of electronic coupling between the two AB moieties occurs in this compound.^53^ Indeed, calculations fully support spectroscopic data, e.g., in **3a** as a pure isomer (*E*_1_*E*_2_), the theoretical UV–vis spectrum (Figure S28) shows a peak centered at 360 nm with high oscillator strength corresponding to a π–π* transition, so it is red-shifted compared to a single azo unit (**1a** at 310 nm and **2a** at 330 nm). Therefore, the two azobenzene subunits of **3a** are π-conjugated and the absorption transitions are red-shifted, in good agreement with the available literature.^53,57^

**Figure 4:**
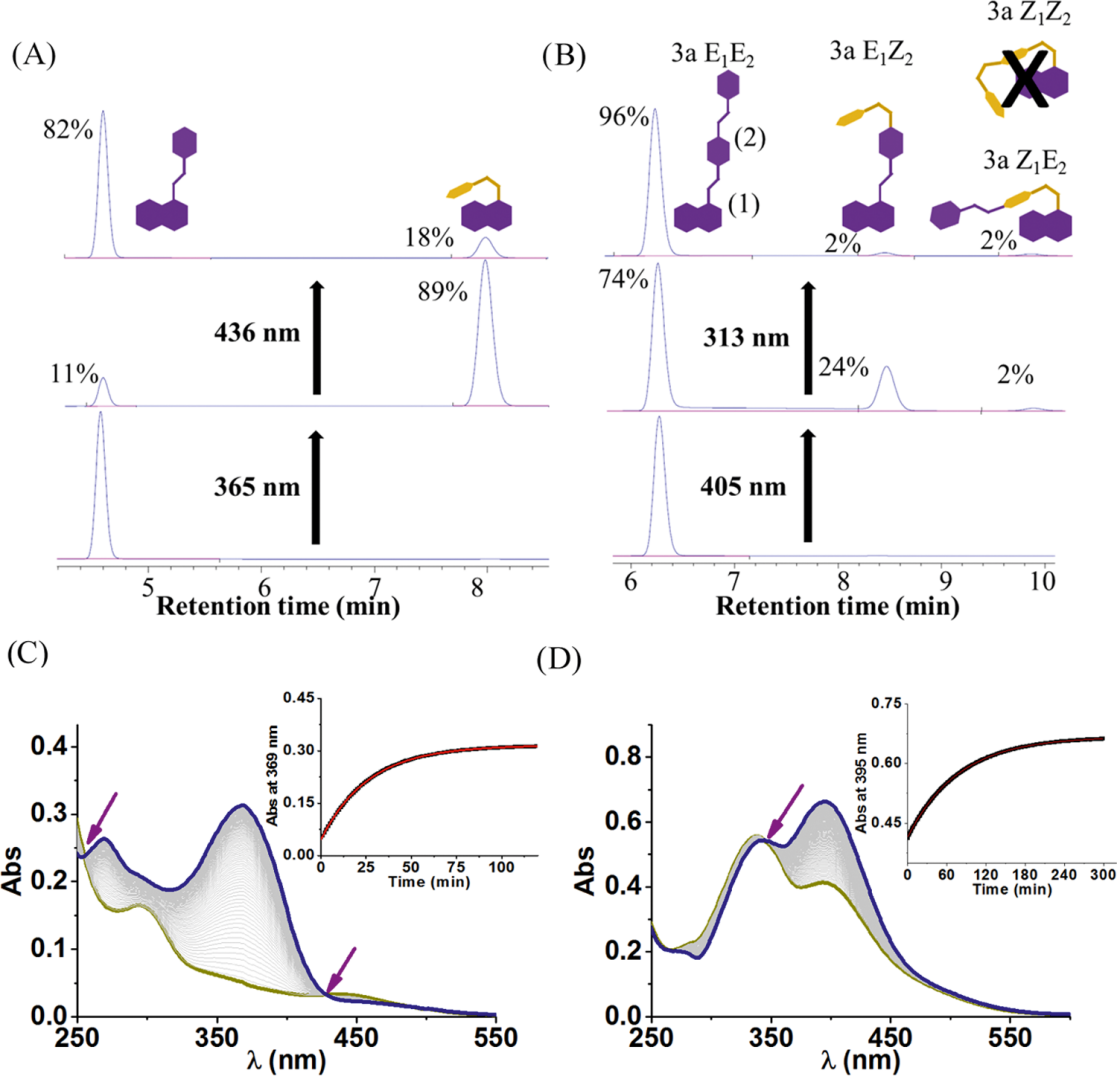
Quantification of the photostationary state of **2a** (A) and **3a** (B) by HPLC analyses with the integration of the UV signal at the wavelengths of the isosbestic points. UV-Vis absorption spectra for 30 uM solution of **2a** at 55 °C (C) and **3a** at 20 °C (D) in acetonitrile in the PSS after 365 nm (C) or 405 nm (D) irradiation (green curve), and spectral evolution during the *Z*–*E* thermal return. The insets present absorption changes during *Z*–*E* thermal return.

The composition of the photostationary state for **1a**, **2a**, and **3a** were derived from HPLC traces (Figures 4A,B and S15). The obtained data show that the PSS of **2a** under 365 nm irradiation is composed of 11/89 *E*/*Z* and 82/18 when under exposure to 436 nm (Figure 4A). Interestingly, as **3a** is noncentrosymmetric with two linked azobenzene motifs, theoretically the mixture of four different geometry compounds is possible. However, as indicated by HPLC, just three states of **3a** are present in the mixture after light exposure (Figure 4B). As the elution of the compounds depends on their polarity, the theoretical calculations have been performed to evaluate the dipole moments of **3a**: *E*_1_*E*_2_, *E*_1_*Z*_2_, *Z*_1_*E*_2_, and *Z*_1_*Z*_2_ to assign the peaks present on the chromatogram to corresponding geometric structures of **3a** (Figure 4B, Table S4). According to the theoretical predictions, the *Z*_1_*Z*_2_-**3a** isomer exhibits the lowest thermodynamic stability (relative energy of 18.18 kcal mol^–1^) together with the highest polarity (4.62 D) of all four isomers and thus is unlikely to be abundantly present and detectable under the considered experimental conditions (Table S4 and S5). Indeed, under the experimental conditions, *Z*_1_*Z*_2_-**3a** was not observed in the mixture (Figure 4B) during the HPLC measurement.

To gain insight into the effect of π-extension on the thermal stability of the *Z*-isomer, the reaction rates of thermal relaxation were investigated for **1a** (reference), **2a**, and **3a** in acetonitrile at four temperatures (see SI pp. S18–S20). The four evolution curves at different temperatures, for each compound, were fitted with exponential decays of the first-order reaction (Figure 4C,D and S16–S18). Importantly, after exposure to 405 nm radiation, the solution of **3a** contained both *E*_1_*Z*_2_ (24%) and *Z*_1_*E*_2_ (2%) with probably different rate constants of isomerization (not investigated here).^53^ The estimated rate constants at different temperatures, Arrhenius and Eyring parameters, result from contributions of both *E*_1_*Z*_2_ and *Z*_1_*E*_2_ reaction rates (Table S1). The data obtained for **1a**, **2a**, and **3a** clearly indicate that π-extension drastically reduces the half-life of the *Z*-isomer, which is calculated to be on the order of days for **1a**, hours for **2a**, and minutes for **3a**, specifically: 322 h for **1a**, 9 h for **2a**, and 0.5 h for **3a** at 25 °C.

**Figure 5:**
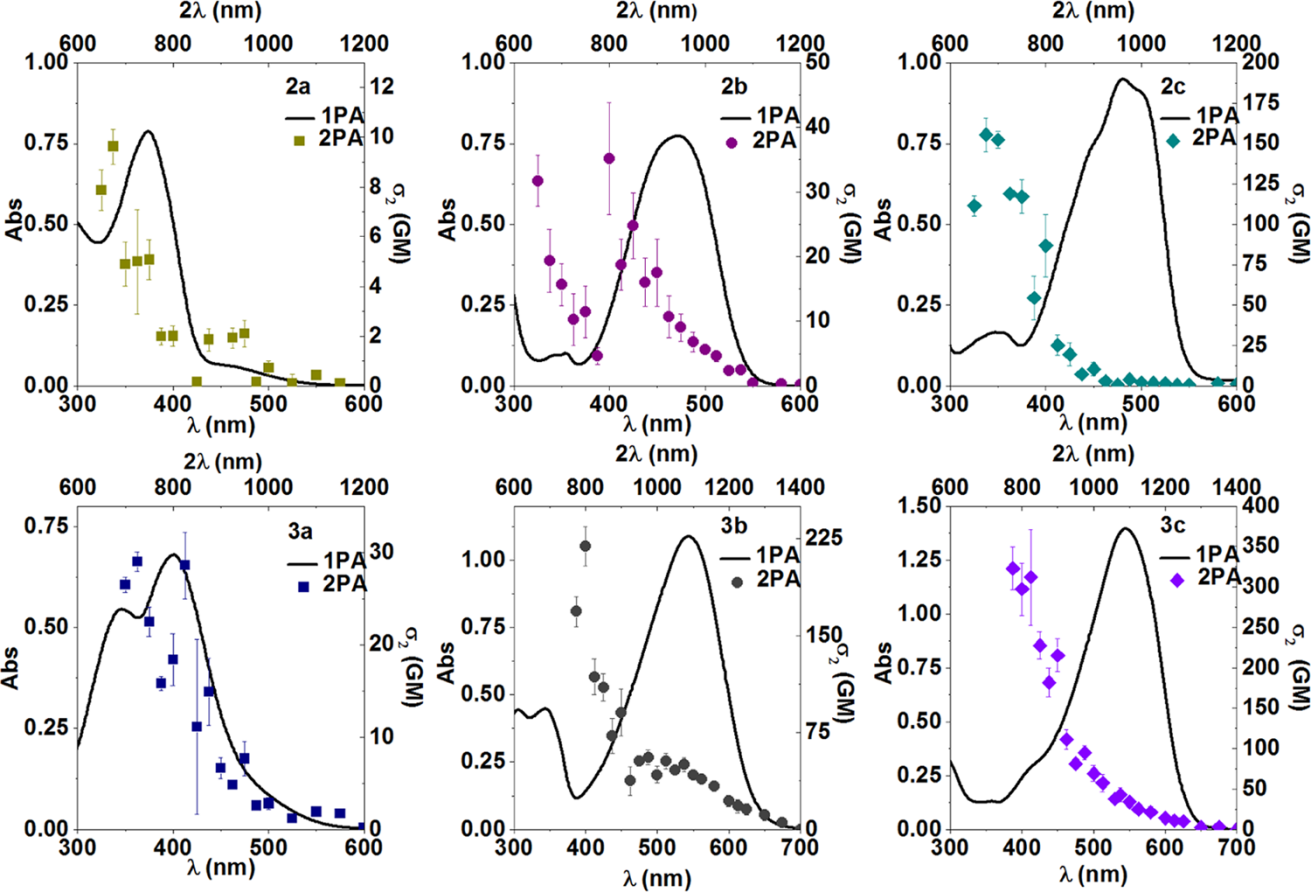
Overlay of one and two-photon absorption spectra for **2a–c** (upper panel) and **3a–c** (lower panel) in DMSO at 20 °C.

Azobenzene derivatives are essentially non-emissive, and thus their 2PA properties could not be investigated by the popular two-photon excited fluorescence technique. Instead, we performed *Z*-scan studies, which rely on power-dependent transmittance measurements. The wavelength range of the measurements was from the tail of their one-photon absorption bands up to 1200 nm, except for **3b** and **3c** which were measured up to 1400 nm. The goal of the studies was to evaluate i) the impact of the length of the π system on 2PA properties, hence we enriched each subsequent series by one (**2a**–**c**) or two (**3a**–**c**) phenyl rings and also ii) the influence of the terminal group(s) on 2PA which was accomplished by synthesizing mono- (**1b**, **2b**, **2c**) and bis- (**1c**, **2c**, **3c**) amino-substituted compounds. The obtained data are presented in Figure 5 and S21, S22, where both 1PA and 2PA spectra are plotted, in the following order: from the left to the right side with increasing number of amino groups, in the lower panel, with an extended π system. Similar to **1b**, **2a**–**3c** are asymmetric, the selection rules do not forbid the one-photon transitions to be two-photon allowed,^36^ on the other hand, **1c** is symmetric and the mutual exclusion principle (one-photon transitions are not two-photon allowed and vice versa) should apply there. Indeed, **1c** does not exhibit notable 2PA at approximately twice 1PA wavelength. Its 2PA maximum is blue-shifted in comparison to 1PA with a 2PA cross-section of about 140 GM at 675 nm in agreement with other results found in the literature (Figure S22).^55,58,59^ The rest of the investigated compounds are noncentrosymmetric, therefore, the S_0_ → S_2_ transition is allowed for both 1PA and 2PA. Indeed, for almost all studied compounds, the two-photon transition can reach the same final state as the one-photon transition (Figures 5 and S22). However, it should be pointed out that in the 2PA spectrum of **2c** and **3c**, there is a low-intensity peak at twice the peak wavelength of the linear absorption, the 2PA maximum is blue-shifted compared to the 1PA maximum. This phenomenon will be explained further in the text, based on theoretical calculations. Moreover, as previously noticed by De Boni and co-workers^58,60,61^ we also observed for some compounds, an increase of the 2PA cross-section when the wavelength gets closer to the region of 1PA, which probably is related to the resonance enhancement of 2PA as the one-photon transition is approached but can also be partly due to the appearance of excited-state absorption in that wavelength region. Moreover, by comparing the upper and lower panels in Figure 5, the influence of the length of the π system (**3a**–**c** enriched with -N=N-Ph) on the 2PA cross-section can be easily assessed. Extension of the π-conjugation enhances the 2PA cross-section by at least a factor of two when comparing **3a**–**c** with **2a**–**c**. However, there is almost no difference in 2PA properties upon the comparison of **1b**-**1c** with **2b**-**2c** (Table 2, Figures 5 and S22). As expected from theory, the 2PA cross-section should depend on the square of the transition dipole moment^36^ (Table 1), therefore, the observed trend can be ascribed to almost negligible changes in the transition dipole moments: 3.49 and 3.46 for **1b** and **2b** respectively; 3.81 and 3.71 for **1c** and **2c** respectively. Additionally, if we analyze Figures 5 and S22 from the left to the right side we will consider three types of systems: π (**2a**, **3a**), D−π (**2b**, **3b**), and D−π–D (**2c**, **3c**). Modification of the central bridge by increasing its accepting ability significantly enhances the 2PA properties (Table 2).

**Table 2:** Concentration, experimental (exp) and theoretical (calc) 2PA properties of azo dyes in DMSO.

cmpd	*C* (mM)	λ_2PA_^exp^^a^ (nm)	σ_2_^exp^^b^ (GM)	σ_2_^exp^^c^	λ_2PA_^calc^^a^ (nm)	σ_2_^calc^ (GM)
**1b**	14.0	800	39 ± 5	0.20	488	136^55^
**1c**	13.0	675	134 ± 6	0.63	541	31^55^
**2a**	12.0	675	6.9 ± 0.7	0.03	566	7
		725	5.0 ± 2.1	0.02	667	33
		950	2.1 ± 0.5	0.01	905	2
**2b**	11.1	800	35 ± 9	0.14	777	59
**2c**	5.20	675	156 ± 11	0.60	598	125
		900	11 ± 3	0.04	792	1
**3a**	12.0				611	607
		725	29 ± 1	0.09	723	51
		825	29 ± 4	0.09	961	16
**3b**	2.00				629	141
		800	219 ± 15	0.62	846	355
		1025	53 ± 6	0.15	925	6
**3c**	1.90				693	1651
		825	312 ± 60	0.85	861	16
		975	95 ± 9	0.26	918	18

a Wavelength of the maximal 2PA value detected.

b Cross-section at maximum.

c GM mol g^–1^.

To investigate the 2PA properties of **2a**–**3c** in more detail we performed DFT calculations. The obtained results, although suffering from the CAM-B3LYP functional error and the arbitrary value of the broadening factor applied in calculations, present the same tendencies as those of experimental *Z*-scan measurements. Overall, the calculations unequivocally confirm the strong increase of the 2PA cross-section upon the introduction of one or two electron-donating amino substituents (from 33 GM for **2a** to 125 GM for **2c**) and by the elongation of the AB scaffold (from 125 GM for **2c** to 1651 GM for **3c**). For **2a** and **2b**, the theoretical data predict the occurrence of the 2PA for the wavelengths corresponding roughly to twice the 1PA wavelength (Figure S31). In the case of **2c**, however, the 2PA activity is observed mostly in the range of weak one-photon absorption, with only small 2PA cross-sections for the wavelengths close to 2λ^1PA^. This intensive 2PA at 598 nm with the 2PA cross-section of about 125 GM involves the transition to the final π* state with the electron density concentrated mostly on the naphthalene moiety (Figure S33). Similar properties of bis-azobenzene derivatives can also be noticed: for **3a** and **3b** the 2PA activity involves the bright 1PA states, however, in **3c** again the strong 2PA signal (1651 GM) appears significantly blue-shifted with respect to the twice 1PA range (Figure S32). On the other hand, only the small cross-sections of about 18 GM are observed for the bright 1PA state at about 908 nm for **3c**. Although the structure of **2c** and **3c** can be perceived as analogous, differing only by the elongation of the π-electron skeleton, the careful analysis of the states involved in 1PA and 2PA transitions (Figure S33) exhibits a significant influence of the naphthalene unit present in the small **2c** scaffold in contrast to its mild effect in a larger **3c** molecule. Since the parent *E*-AB molecule is assumed to exhibit C_2h_ symmetry, as confirmed both in the gas electron diffraction experiment and theoretical calculations,^62−65^ the **2c** and **3c** systems can be perceived as C_2h_-like, due to the presence of the AB scaffold with two amino substituents in *para* positions. The distortion to this symmetry point group is introduced by the presence of the second aromatic ring (naphthalene unit instead of phenyl) and by the pyramidalization of amino groups (see Supporting Information p. S25). Thus, according to the Laporte rule, one could expect that 1PA should occur with the change of parity of the states, while the 2PA should be active for states conserving parity. Indeed, in the case of **3c**, the 1PA-allowed transition is observed with the change of the parity to the final S_3_ state of the π* character, in agreement with the selection rule, while the 2PA-allowed transition to the π* S_4_ state maintains the symmetry of the involved states (Figures S33 and S34 and Tables S6, S7). This provides evidence for the fact that the symmetry of the excited-state is only subtly perturbed by the abovementioned structural modifications in **3c**, based on the visual analysis of molecular orbitals. Yet, in the case of **2c**, both 1PA and 2PA-allowed transitions occur with the change of parity, which indicates that the presence of the additional aromatic ring significantly disturbs the ideal symmetry for this smaller molecule.

## Conclusions

In summary, we have explored a molecular design-based strategy to optimize the 2PA properties of mono- and bis-azobenzene derivatives substituted with amino group(s). Our approach is based on: (i) π-conjugation extension and (ii) introduction of EDG(s) into the structure of the dyes. To verify our approach three series of systems (nine compounds) have been designed and synthesized: π (**1a**, **2a**, **3a**), D−π (**1b**, **2b**, **3b**), and D−π–D (**1c**, **2c**, **3c**) systems including mono- (**1a**–**2c**) and bis-azobenzene (**3a**–**c**) derivatives and their photochromic behavior and 2PA properties were studied. To understand the experimental findings more deeply, DFT calculations have been performed.

We have shown that, as the π-conjugation length increases, the π–π* S_0_ → S_2_ vertical transitions are red-shifted and a significant decrease in the half-life of the *Z*-isomer at room temperature is observed, changing from days (**1a**) to minutes (**3a**). Due to the electronic conjugation of two azobenzene units in **3a**, a poorer *Z*-content at PSS_313 nm_ can be achieved compared to single azobenzene compounds (**1a**, **2a**). However, we expect that the *Z*-isomer content at PSS could be improved by electronic decoupling of two azobenzene motifs, i.e., by the incorporation of an additional phenyl ring between the azo bridges. Moreover, photoswitching of **3a** may lead to four isomers in the PSS mixture, however, just three photoisomers were detected in the solution. With the help of DFT calculations, we confirmed that **3a**-*Z*_1_*Z*_2_ should not appear in the solution after illumination as this isomer has the lowest stability (Table S4) and the highest dipole moment (should be eluted as the last one in HPLC).

Furthermore, the present investigation points to the importance of extension of the π-conjugation for the 2PA cross-sections. To account for the variation in the sizes of the respective molecules, the comparisons can be carried out in terms of the molecular weight normalized cross-sections, σ_2_/M. In the sequence: **2a** → **3a**, values of 0.03 GM·mol·g^–1^ and 0.09 GM·mol·g^–1^; **2b** → **3b**, values of 0.14 GM·mol·g^–1^ and 0.62 GM·mol·g^–1^; and **2c** → **3c**, values of 0.60 GM·mol·g^–1^ and 0.85 GM·mol·g^–1^ are obtained, respectively. Finally, a further enhancement of the 2PA cross-section was achieved by proceeding to the introduction of terminal EDG(s), by almost 20 times when comparing **2a** with **2c** and 10 times when juxtaposing **3a** with **3c**. Experimentally, the 2PA maxima for **1c**–**3c** were blue-shifted with respect to the 1PA maxima, which is puzzling for **2c** and **3c** (noncentrosymmetric compounds). These features were explained by DFT calculation which anticipates the presence of 2-photon active excited states at higher energies, not at twice the wavelength of 1PA. Moreover, DFT calculations allowed us to gain insight into the 1- and 2-photon electronic transitions of azobenzene derivatives, corroborating the interpretation of the observed experimental features. The most intriguing theoretical results arise, however, from the symmetry considerations for the involved excited states. Based on visual analysis of molecular orbitals, it can be noticed that the perturbation of symmetry is less pronounced for **3c** than for its smaller analogue **2c**. The importance of symmetry breaking for the conservation of the selection rules in the case of smaller systems in contrast to bis-azobenzene molecules can be vital for the further controlled design of the strong 2PA absorbers and fine-tuning of their nonlinear optical features.

The present work is one of few attempts made up to now to investigate the linear and nonlinear properties of the photochromic compounds in a systematic way and can serve as a platform for the future design of azo compounds with desired properties. Moreover, according to our knowledge, it is the first study of the bis-azobenzene molecules in the context of their 2PA efficiency in a wide spectral range.

## Supporting Information Available

Synthetic details and compound characterization, photoisomerization studies, as well as thermal stability of the synthesized azobenzene derivatives, and computational details and results (PDF)
