# Supplementary material for: Linear and Nonlinear Optical Properties of Azobenzene Derivatives Modified with an (Amino)naphthalene Moiety
Source: J Phys Chem B. 2022 Aug 9;126(32):6063–73. doi: 10.1021/acs.jpcb.2c03078 (PMC9393860; doi:10.1021/acs.jpcb.2c03078)
Supplement: Supplementary file 1 — jp2c03078_si_001.pdf [file jp2c03078_si_001.pdf]

SUPPORTING INFORMATION

Linear and Nonlinear Optical Properties of Azobenzene Derivatives Modified with (Amino)naphthalene Moiety

Marta Dudek,[a] Anna Kaczmarek-Kędziera,[b] Radosław Deska,[a] Jakub Trojnar,[a] Patryk Jasik[c] Piotr Młynarz,[d] Marek Samoć[a] and Katarzyna Matczyszyn[a]*

[a] Advanced Materials Engineering and Modelling Group, Faculty of Chemistry, Wrocław University of Science and Technology, Wyb. Wyspiańskiego 27, 50-370 Wrocław, Poland

[b] Faculty of Chemistry, Nicolaus Copernicus University in Toruń, Gagarina 7, 87-100 Toruń, Poland

[c] Faculty of Applied Physics and Mathematics and BioTechMed Center, Gdańsk University of Technology, Gabriela Narutowicza 11/12, 80-233 Gdańsk, Poland

[d] Department of Biochemistry, Molecular Biology and Biotechnology, Faculty of Chemistry, Wrocław University of Science and Technology, Wyb. Wyspiańskiego 27, 50-370 Wrocław, Poland

Corresponding Author
*E-mail: marta.ziemianek-dudek@pwr.edu.pl

Table of Contents

1. Synthetic and Analytical General Methods S2
2. Synthesis and characterization S2
3. Photoisomerization studies S17
4. Thermal Stability S18
5. Z-scan studies S21
6. Theoretical calculations S22
7. References S35

1. SYNTHETIC AND ANALYTICAL GENERAL METHODS

Solvents and starting materials were purchased from commercial suppliers and were used as received. Reactions were monitored by thin layer chromatography (TLC) carried out on silica gel plates (Merck 60F-254). Column chromatography was performed using silica gel (Merck 60, particle size 0.040–0.063 mm). NMR-spectra were recorded on a Bruker AvanceTM 600 MHz spectrometer or on a JEOL 400 MHz spectrometer at 25°C using residual protonated solvent signals as internal standards for ^{1}H-and ^{13}C-spectra. Multiplicities are abbreviated as follows: singlet (s), doublet (d), triplet (t), quartet (q), multiplet (m), and broad (br). High resolution mass spectra (HRMS) were conducted with a WATERS LCT Premier XE mass spectrometer (ESI). High Pressure Liquid Chromatography (HPLC) was done using an Agilent 1260 Infinity II equipped with a chiralpak IB column in hexane/isopropanol.

2. SYNTHESIS AND CHARACTERIZATION

General procedure for azo coupling reaction[1]

Aniline derivative (1equiv) was added into the mixture of H_2O/EtOH (2/1) and HCl (2 equiv) at 0˚C soon afterward dropwise addition of cold water solution of $NaNO_2$ (1 equiv) over a period of 10 minutes, then the mixture was stirred for 1 h at 0˚C. After that time, the reaction mixture was neutralized to pH = 6 using potassium acetate. Next, the diazonium salt solution was added dropwise into the solution of 1-naphthylamine (1equiv) and HCl (1equiv) at H_2O/EtOH (2:1). The suspension was left stirring for next 1 h at 0˚C and left overnight at room temperature. The solution was then neutralized with addition of ammonium solution (25%). Crude product was purified by column chromatography to give the corresponding azobenzene derivative.

General procedure for oxidation of aniline to nitrosoarene derivatives[2]

To a solution of the aniline derivative (1 equiv) in DCM was added the solution of Oxone (2 equiv) in water. The mixture was stirred at room temperature until the reaction was completed (progress of the reaction was monitored by TLC). Then, two phases were separated and the organic phase was washed with water, dried over $MgSO_4$, filtered, and the solvent was removed under reduced pressure. The resulting nitroso derivatives were used without further purification in the following Mills reaction.

General procedure for Mills reaction[3]

To a solution of the aniline derivative (1equiv) in acetic acid was added the nitrosoarene (1equiv) and the resulting mixture was stirred overnight at room temperature. Then the obtained precipitate was filtered and washed thoroughly with water. The resulting solid was purified by column chromatography to give the corresponding azobenzene derivative.

General procedure for reduction of diazonium salts[4]

A solution of amine derivative (1 equiv) and HCl (1 equiv) in H_2O/EtOH (2:1) was chilled in an ice-salt bath and treated with cold water solution of sodium nitrate (1 equiv). The resulting solution was stirred for 30 min, and the hypophosphorous acid (50%, 20 equiv) was added dropwise over 10 min. The resulting mixture was left stirring at 0-5˚C for 2 h and then left overnight at room temperature. The resulting mixture was neutralized with ammonium solution (25%) and extracted with EtOAc (2x50 mL), washed with water and brine, dried over $MgSO_4$, filtered and concentrated under reduced pressure. The resulting solid was purified by column chromatography to give the corresponding azobenzene derivative (**2a**, **3a**).

Synthesis of 1a-1c

Compound **1b** was purchased from Sigma-Aldrich and compound **1c** was synthesized according to previously published procedure.[5]

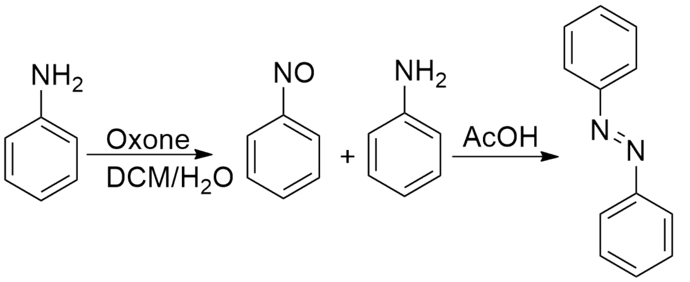

1a

Scheme S1. Synthetic route for **1a**.

Nitrosobenzene: Standard oxidation of aniline to nitrosoarene derivatives procedure was used with aniline (1.20 g, 12.9 mmol) in DCM (40 mL) and Oxone (7.92 g, 25.8 mmol) in H_2O (120 mL) to give **nitrosobenzene** as brown crystals that was used as such in the next step.

1a: Standard Mills reaction procedure was used with **nitrosobenzene** (1.38 g, 12.9 mmol) and aniline (1.20 g, 12.9 mmol) in AcOH (70 mL). The residue was purified by column

chromatography using as eluent pure hexanes to DCM/hexanes 30/70 to yield **1** (1.34 g, 57%) as an orange solid. **^{1}H NMR** (601 MHz, Chloroform-d) δ: 7.95 – 7.92 (m, 4H), 7.55 – 7.51 (m, 4H), 7.50 – 7.45 (m, 2H). **HRMS** m/z (ESI): $C_{12}H_{10}N_2$ $[M+H]^+$,calculated: 183.0922, found:183.0917.

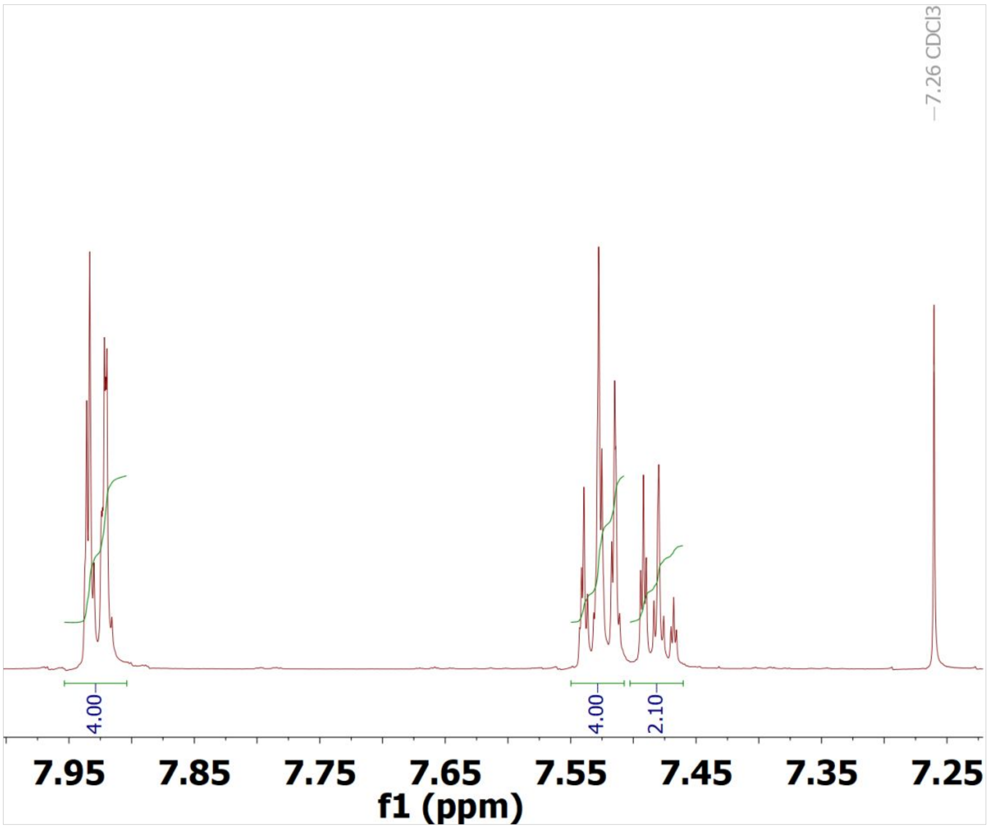

Figure S1. ^{1}H NMR spectrum of **1a**.

Synthesis of 2a-2c

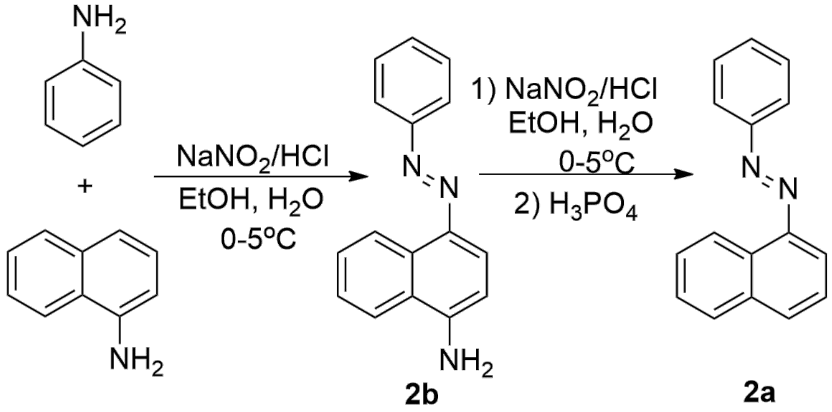

Scheme S2. Synthetic route for **2a** and **2b**

2b: Standard azo coupling procedure was used with aniline (3.0 g, 32.2 mmol) in H_2O/EtOH (20/10 mL), HCl (10 M, 6.4 mL), $NaNO_2$ (2.2 g, 32.2 mmol) and then 1-naphtylamine (4.6 g, 32.2 mmol) in H_2O/EtOH (75/25 mL), HCl (10 M, 3.2 mL). The residue was purified by column chromatography using as eluent hexanes/CH_2Cl_2 1/1 to pure CH_2Cl_2 to yield **2b** (4.8 g, 60%) as a red solid. **^{1}H NMR** (601 MHz, Methylene Chloride-d_2) δ: 9.11 (d, J = 8.5 Hz, 1H), 8.04 (d, J = 7.7 Hz, 2H), 7.98 (d, J = 8.3 Hz, 1H), 7.83 (d, J = 8.5 Hz, 1H), 7.73 – 7.67 (m, 1H), 7.61 – 7.54 (m, 3H), 7.48 (t, J = 7.3 Hz, 1H), 6.82 (d, J = 8.3 Hz, 1H), 4.70 (s, 2H). **^{13}C NMR** (151 MHz, Methylene Chloride-d2) δ: 153.6, 146.8, 140.0, 133.2, 129.8, 129.1, 127.2, 125.3, 123.9, 122.6, 122.4, 120.8, 114.0, 108.9. **HRMS** m/z (ESI): $C_{16}H_{13}N_3$ $[M+H]^+$, calculated: 248.1188, found: 248.1186.

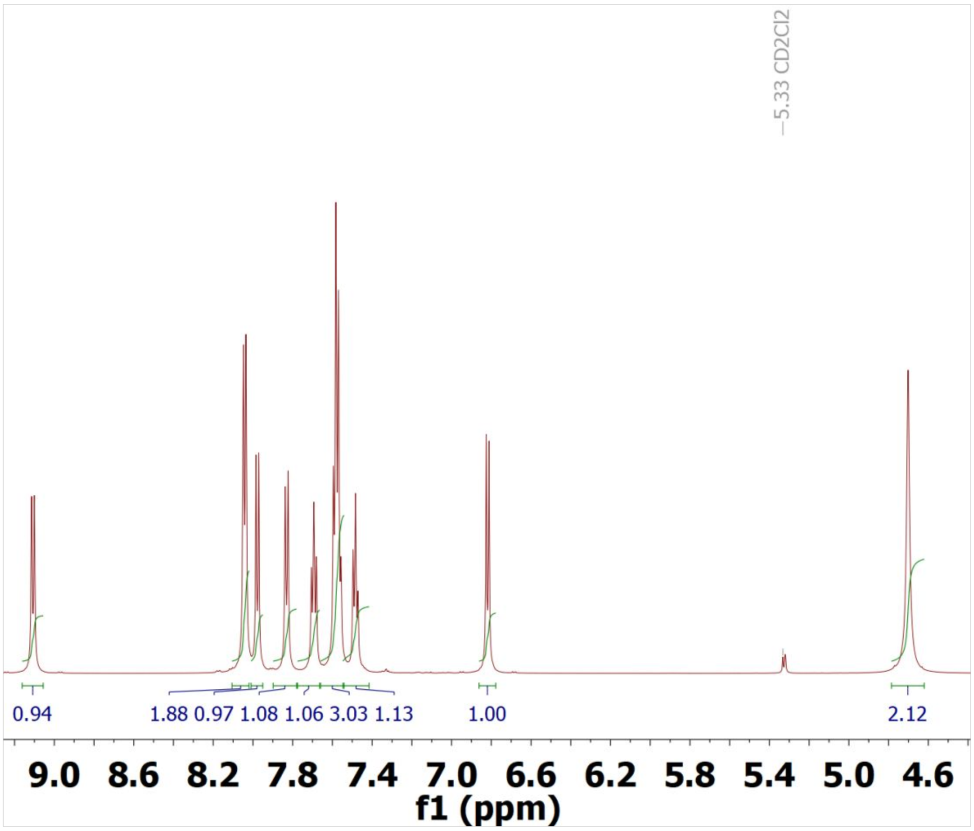

Figure S2. ^{1}H NMR spectrum of **2b**.

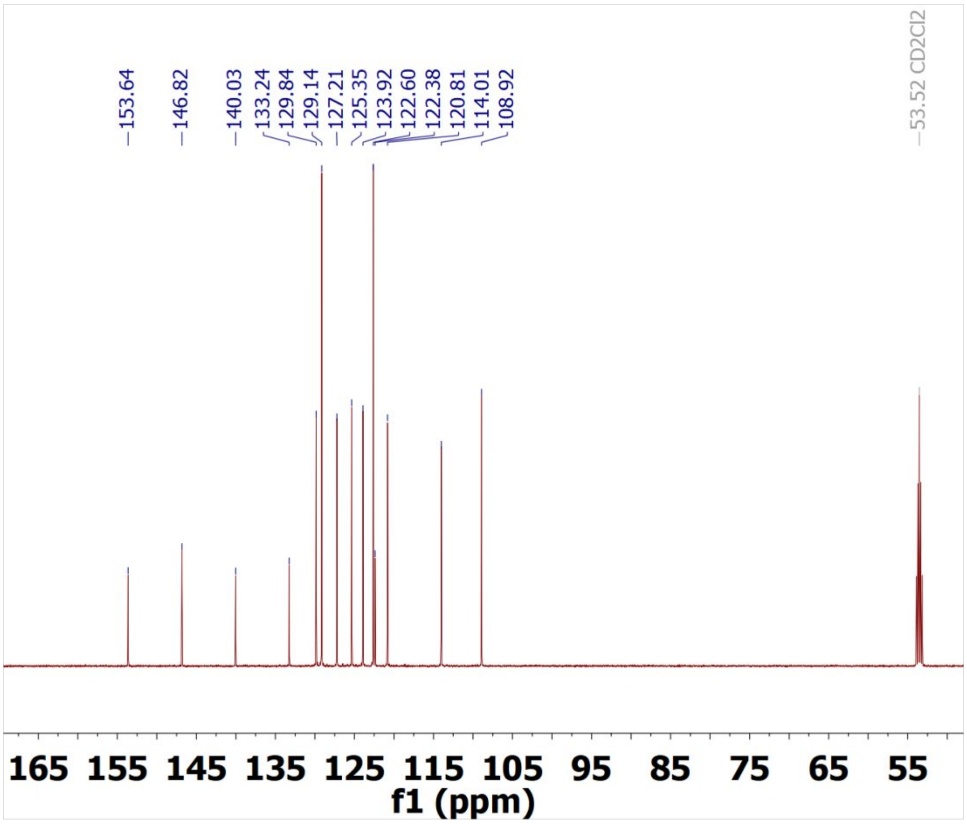

Figure S3. ^{13}C NMR spectrum of **2b**.

2a: Standard reduction of diazonium salts procedure was used with **2b** (0.3 g, 1.21 mmol) in H_2O/EtOH (8/4 mL), HCl (10 M, 1.2 mL), $NaNO_2$ (83.5 mg, 1.21 mmol) and H_3PO_2 (50%, 1.07 mL). The residue was purified by column chromatography using as eluent pure hexanes to hexanes/CH_2Cl_2 90/10 to yield **2a** (165 mg, 59%) as a red solid. **^{1}H NMR** (601 MHz, Methylene Chloride-d_2) δ: 8.94 (d, J = 8.4, 1.1 Hz, 1H), 8.08 – 8.04 (m, 2H), 8.03 (d, J = 8.1 Hz, 1H), 7.97 (d, J = 8.2 Hz, 1H), 7.84 (dd, J = 7.5, 1.1 Hz, 1H), 7.70 – 7.64 (m, 1H), 7.64 – 7.56 (m, 4H), 7.57 – 7.51 (m, 1H). **^{13}C NMR** (151 MHz, Chloroform-d) δ 153.2, 147.8, 134.4, 131.4, 131.1, 129.2, 128.0, 126.9, 126.5, 125.7, 123.5, 123.2, 111.9. **HRMS** m/z (ESI): $C_{16}H_{12}N_2$ [M+H]$^+$, calculated: 233.1079, found: 233.1077.

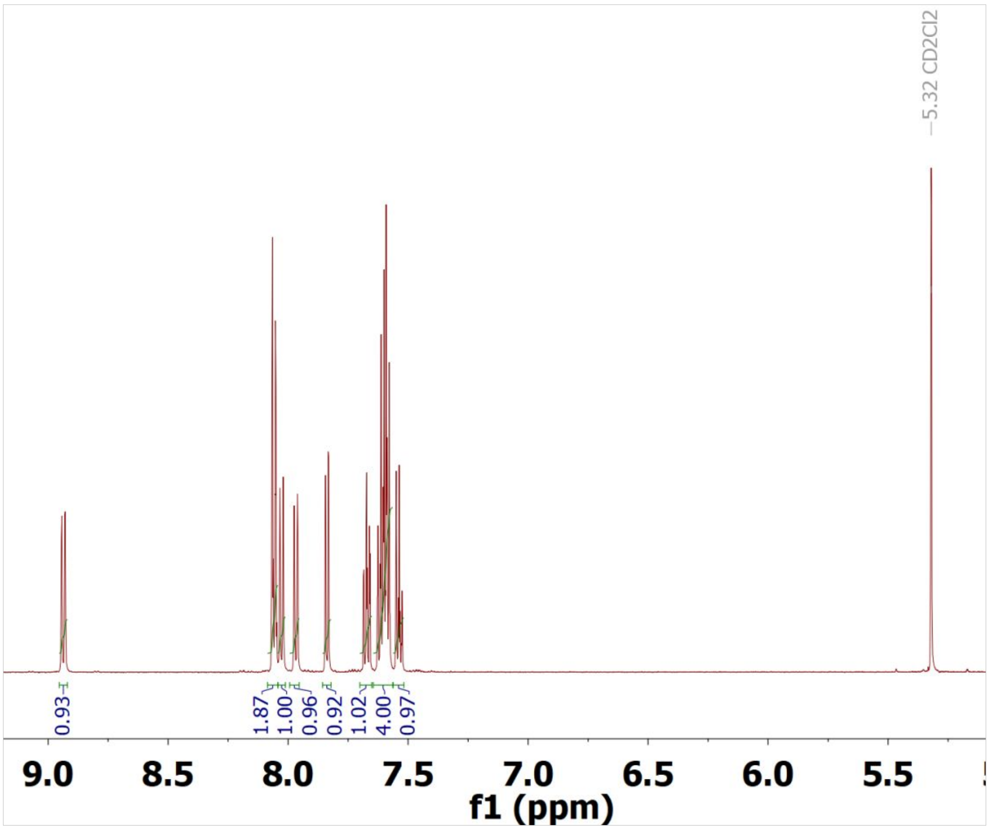

Figure S4. ^{1}H NMR spectrum of **2a**.

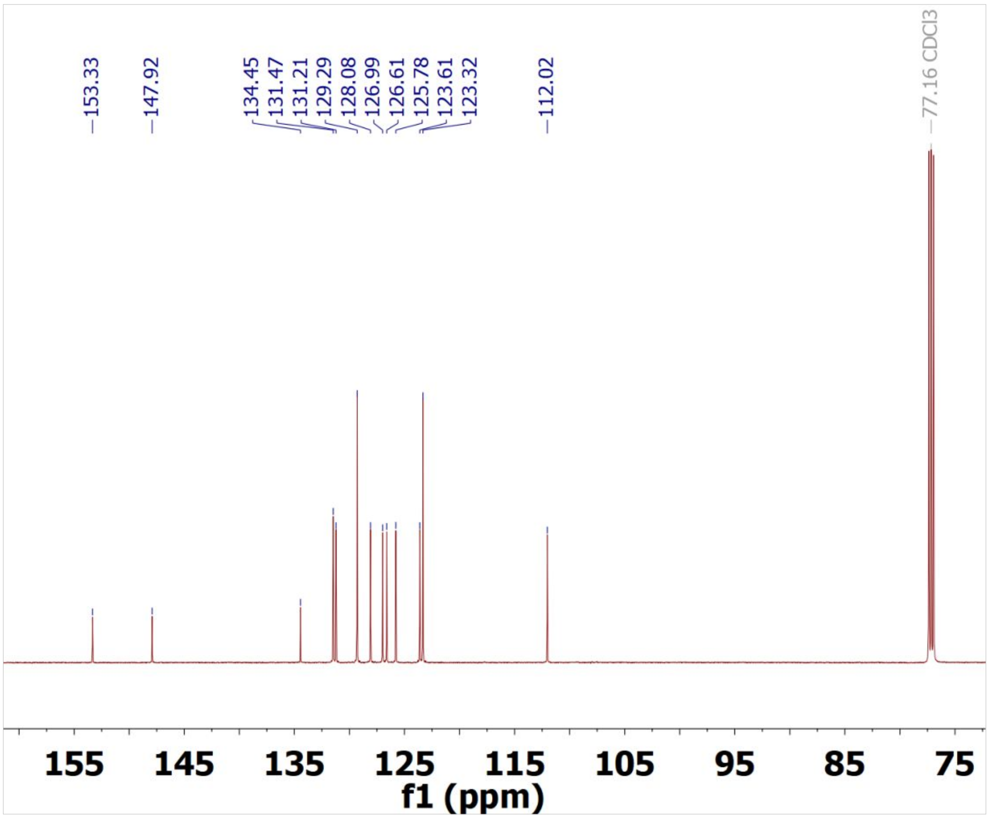

Figure S5. ^{13}C NMR spectrum of **2a**.

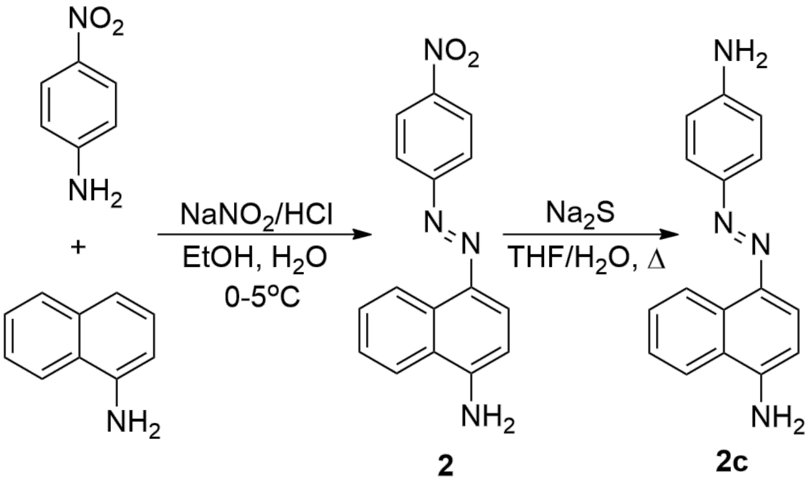

Scheme S3. Synthetic route for **2c.**

2: Standard azo coupling procedure was used with 4-nitroaniline (1.38 g, 10 mmol) in H_2O/EtOH (20/10 mL), HCl (10 M, 2.0 mL), $NaNO_2$ (0.69 g, 10 mmol) and then 1-naphtylamine (1.43 g, 10 mmol) in H_2O/EtOH (20/10 mL), HCl (10 M, 1.0 mL). The residue was purified by column chromatography using as eluent pure hexanes to hexanes/CH_2Cl_2 50/50 to yield **2** (0.94 g, 32%) as a purple solid. **^{1}H NMR** (400 MHz, Methylene Chloride-d_2) δ: 9.05 (d, J = 8.0 Hz, 1H), 8.42 – 8.30 (m, 2H), 8.10 – 8.03 (m, 3H), 7.87 (d, J = 8.5 Hz, 1H), 7.74 –

7.67 (m, 1H), 7.64 – 7.54 (m, 1H), 6.86 (d, *J* = 8.5 Hz, 1H), 4.98 (br s, 2H). **HRMS** m/z (ESI): $C_{16}H_{12}N_4O_2$ $[M+H]^+$,calculated: 293.1039, found: 293.1043.

2c: To a solution of **2** (0.16 g, 0.55 mmol, 1 equiv) in H_2O/THF 1:3 v/v (40 mL), Na_2S (0.396 g, 1.65 mmol, 3 equiv) was added and the mixture was refluxed for 4 h. The THF was evaporated and residues were diluted with 1 M NaOH and extracted with EtOAc (3 x 50 mL). Layers were separated and organic layers were combined, washed with brine and dried over $MgSO_4$. After evaporation of the solvent, crude product was purified by column chromatography on SiO_2 gradient from $CHCl_3$ to 99/2 $CHCl_3$/MeOH to give product as deep red powder (0.12 g, 82%). **^{1}H NMR** (400 MHz, Methylene Chloride-d_2) δ: 9.00 (d, *J* = 8.4 Hz, 1H), 7.89 – 7.78 (m, 4H), 7.66 – 7.60 (m, 1H), 7.58 – 7.51 (m, 1H), 6.83 (d, *J* = 8.3 Hz, 1H), 6.81 – 6.74 (m, 2H), 4.60 (s, 2H), 4.08 (s, 2H). **^{13}C NMR** (151 MHz, Methylene Chloride-d_2) δ: 149.0, 146.3, 145.3, 140.3, 132.7, 126.6, 125.1, 124.5, 124.0, 122.6, 120.7, 114.6, 113.1, 109.1. **HRMS** m/z (ESI): $C_{16}H_{14}N_4$ $[M+H]^+$,calculated: 263.1297, found: 263.1295.

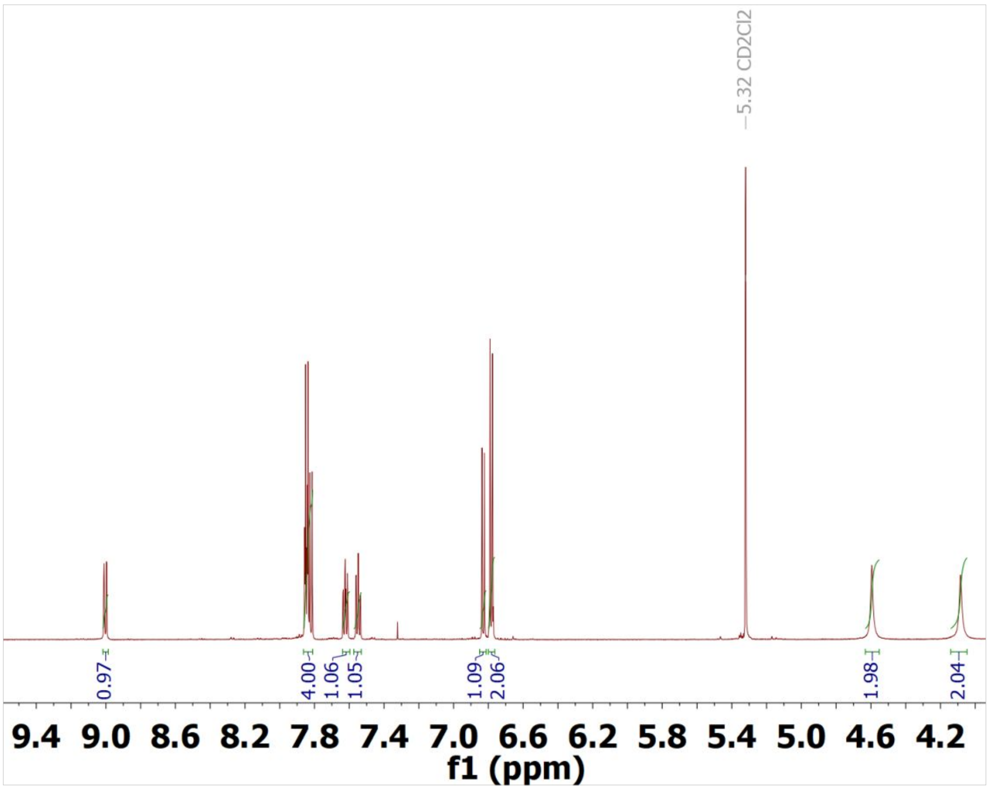

Figure S6. ^{1}H NMR spectrum of **2c**.

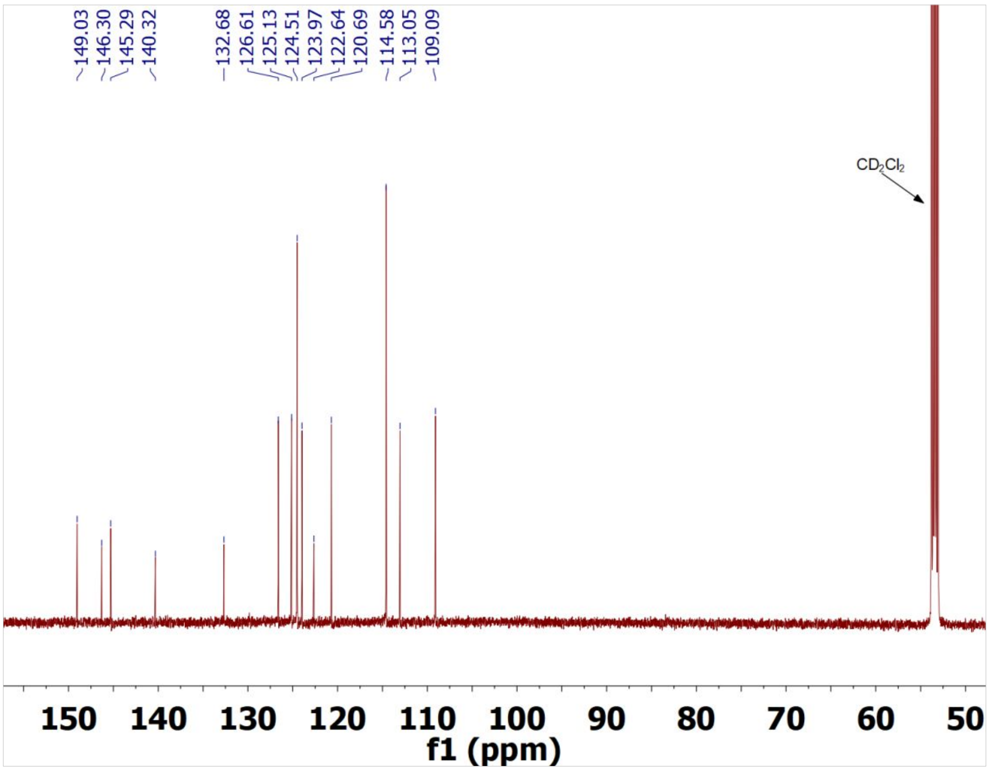

Figure S7. ^{13}C NMR spectrum of **2c**.

Synthesis of 3a-3c

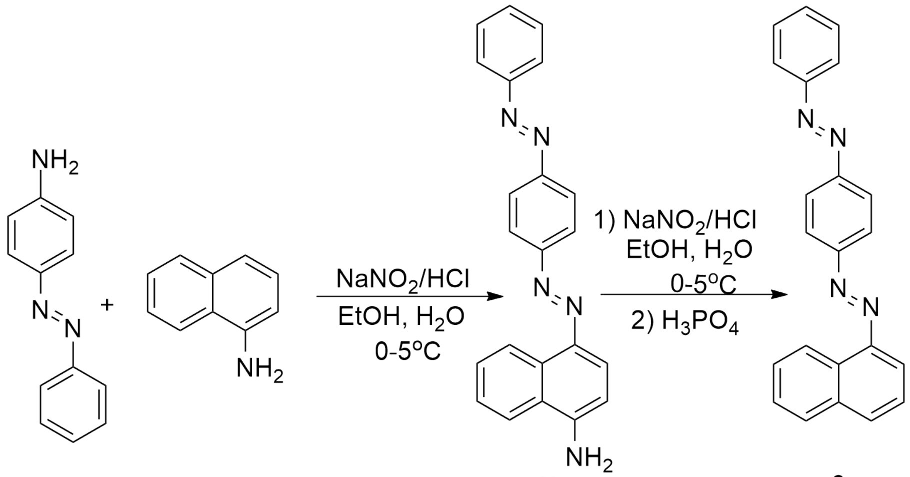

Scheme S4. Synthetic route for **3a** and **3b.**

3b: Standard azo coupling procedure was used with 4-aminoazobenzene (1.97 g, 10 mmol) in H_2O/EtOH (30/15 mL), HCl (10 M, 2 mL), $NaNO_2$ (0.69 g, 10 mmol) and then 1-naphtylamine (1.43 g, 10 mmol) in H_2O/EtOH (20/10 mL), HCl (10 M, 1.0 mL). The residue

was purified by column chromatography using as eluent pure hexanes to hexanes/CH_2Cl_2 50/50 to yield **3b** (2.53 g, 72%) as dark purple solid. **^{1}H NMR** (601 MHz, Methylene Chloride-d_2) δ: 9.10 (d, J = 8.5 Hz, 1H), 8.15 – 8.12 (m, 2H), 8.12 – 8.09 (m, 2H), 8.02 (d, J = 8.3 Hz, 1H), 8.00 – 7.97 (m, 2H), 7.85 (d, J = 8.4 Hz, 1H), 7.73 – 7.67 (m, 1H), 7.61 – 7.46 (m, 4H), 6.84 (d, J = 8.3 Hz, 1H), 4.82 (s, 2H). **^{13}C NMR** (151 MHz, Methylene Chloride-d2) δ: 155.5, 153.4, 153.4, 148.0, 140.8, 134.0, 131.8, 129.7, 128.0, 126.0, 124.4, 124.4, 124.0, 123.5, 122.8, 121.3, 115.00, 109.5. **HRMS** m/z (ESI): $C_{22}H_{17}N_5$ $[M+H]^+$,calculated: 352.1562, found: 352.1563.

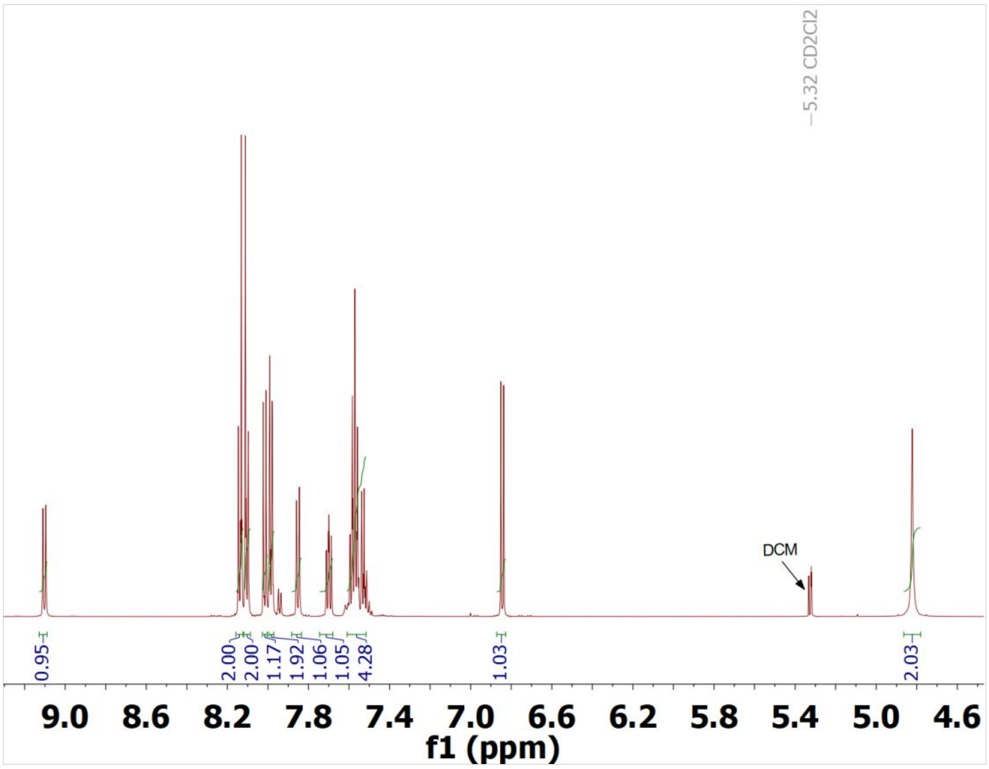

Figure S8. ^{1}H NMR spectrum of **3b**.

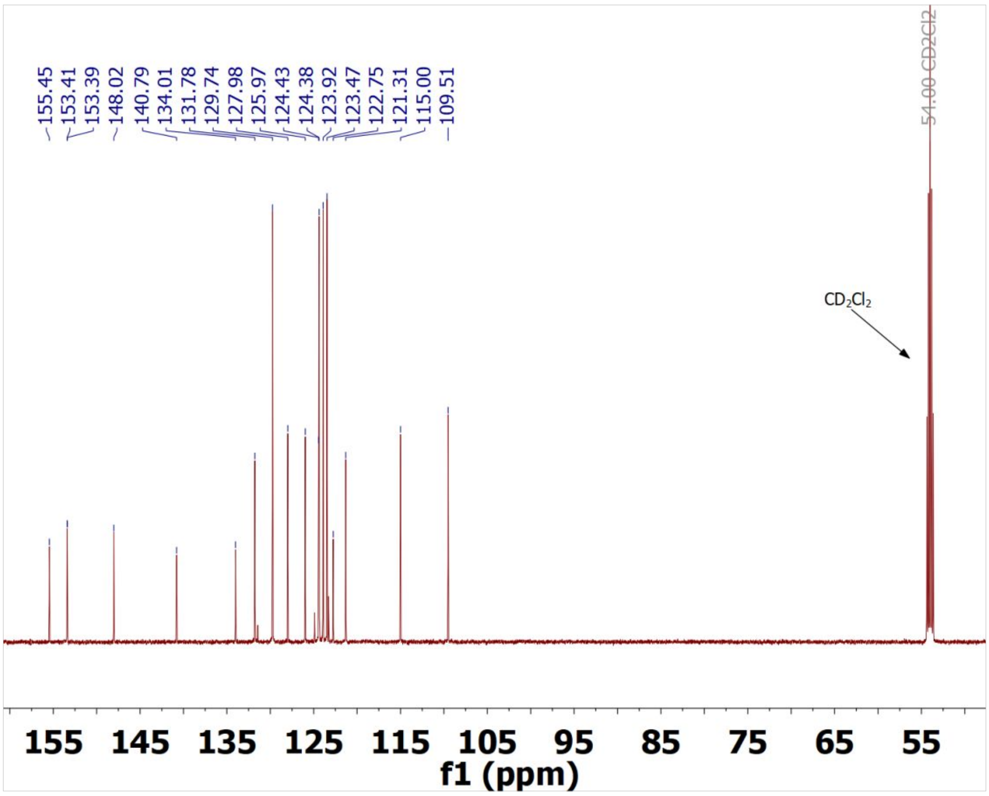

Figure S9. ^{13}C NMR spectrum of **3b**.

3a: Standard reduction of diazonium salts procedure was used with **3b** (0.17 g, 0.48 mmol) in H_2O/EtOH (4/2 mL), HCl (10 M, 0.96 mL), $NaNO_2$ (33 mg, 0.48 mmol) and H_3PO_2 (50%, 0.43 mL). The residue was purified by column chromatography using as eluent pure hexanes to hexanes/CH_2Cl_2 70/30 to yield **3a** (108 mg, 67%) as a red/orange solid. **^{1}H NMR** (400 MHz, Methylene Chloride-d_2) δ: 8.99 (d, J = 8.4 Hz, 1H), 8.22 – 8.20 (m, 2H), 8.15 – 8.12 (m, 2H), 8.06 (d, J = 8.1 Hz, 1H), 8.04 – 7.94 (m, 3H), 7.91 (d, J = 7.5 Hz, 1H), 7.77 – 7.66 (m, 1H), 7.68 – 7.49 (m, 5H). **^{13}C NMR** (151 MHz, Methylene Chloride-d_2) δ 154.8, 154.3, 153.3, 148.3, 135.0, 132.5, 132.1, 132.0, 129.8, 128.6, 127.7, 127.2, 126.2, 124.6, 124.4, 123.9, 123.6, 112.5. **HRMS** m/z (ESI): $C_{22}H_{16}N_4$ $[M+H]^+$,calculated: 337.1453, found: 337.1453.

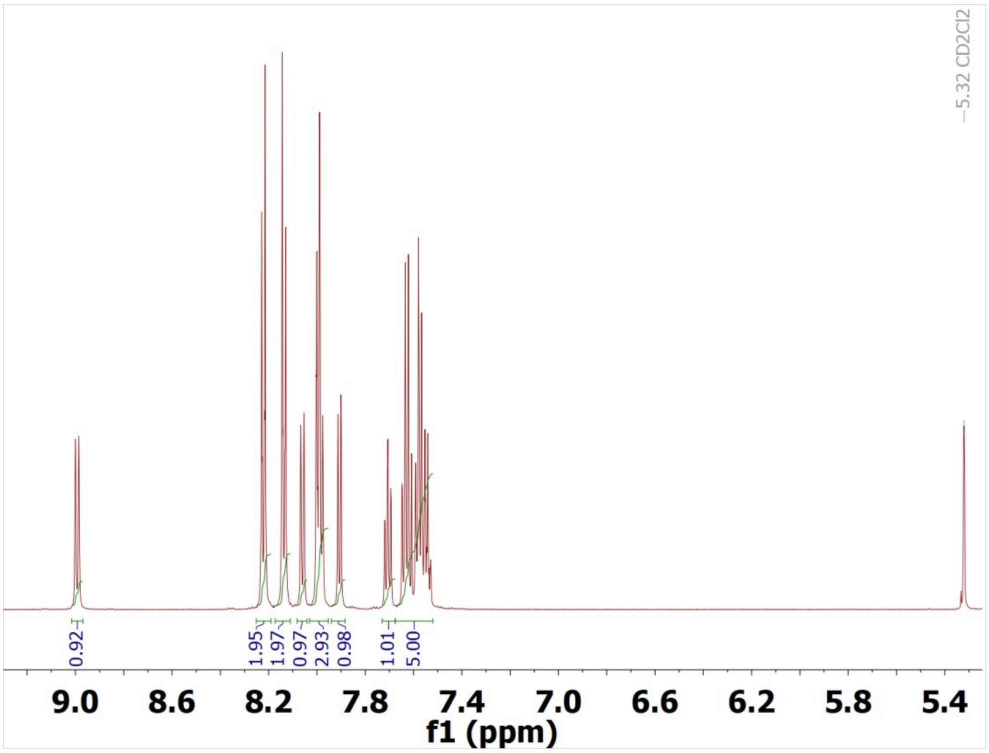

Figure S10. ^{1}H NMR spectrum of **3a**.

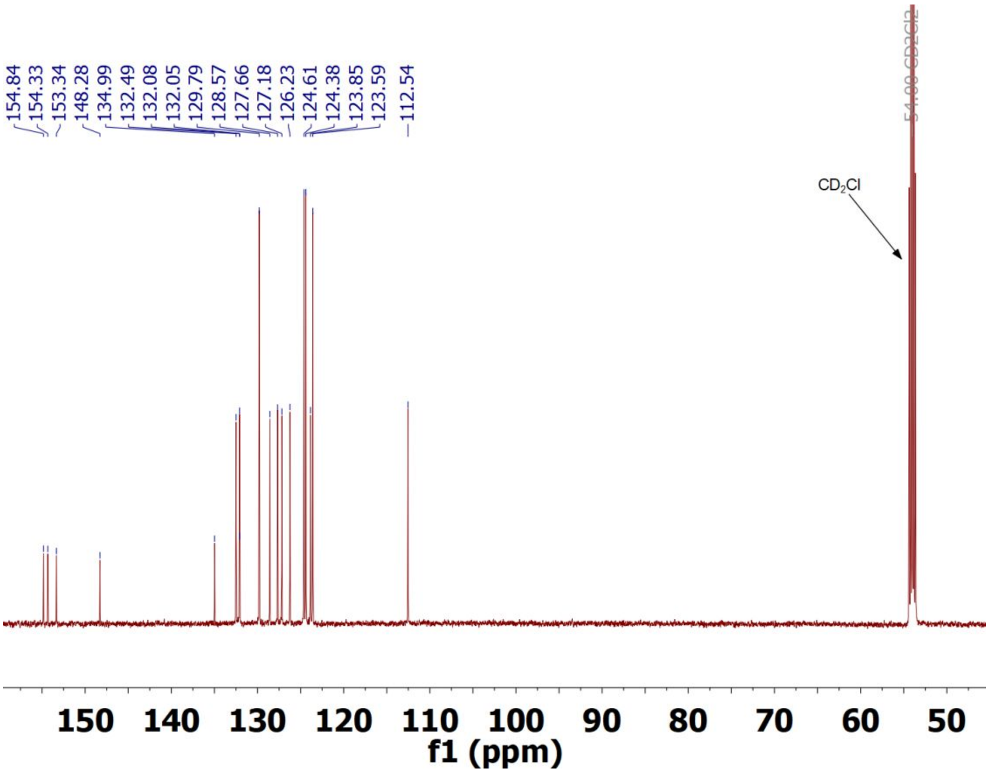

Figure S11. ^{13}C NMR spectrum of **3a**.

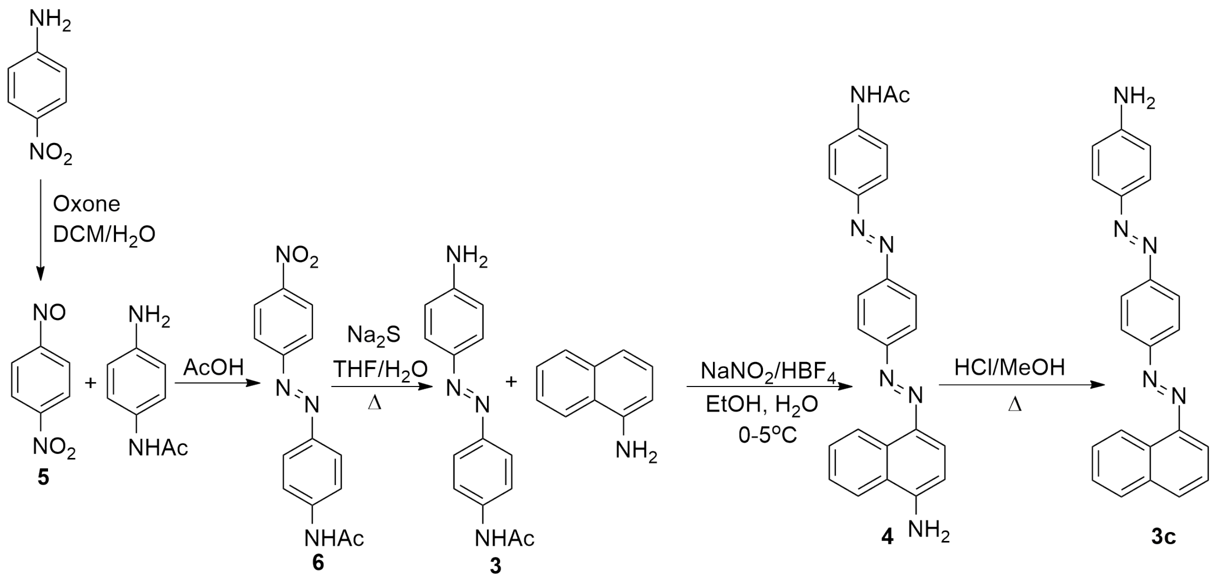

Scheme S5. Synthetic route for **3c.**

5: Standard oxidation of aniline to nitrosoarene derivatives procedures was used with 4-nitroaniline (1.45 g, 10.5 mmol) in DCM (25 mL) and Oxone (6.17 g, 20.1 mmol) in H_2O (75 mL) to give **5** as a deep green liquid that was used as such in the next step.

6: Standard Mills reaction procedure was used with **5** (1.6 g, 10.5 mmol) and 4-aminoacetanilide (1.58 g, 10.5 mmol) in AcOH (70 mL). The residue was purified by column chromatography using as eluent pure DCM to DCM/MeOH 97/3 to yield **6** (2.3 g, 77%) as a red solid. **^{1}H NMR** (400 MHz, Acetone-d6) δ: 9.57 (s, 1H), 8.51 – 8.38 (m, 2H), 8.16 – 8.03 (m, 2H), 8.01 – 7.94 (m, 2H), 7.93 – 7.85 (m, 2H), 2.15 (s, 3H). **HRMS** m/z (ESI): $C_{14}H_{12}N_4O_3$ $[M+H]^+$,calculated: 285.0988, found: 285.0986.

3: To a solution of **6** (0.83 g, 2.9 mmol, 1 equiv) in H_2O/THF 1:3 v/v (90 mL), Na_2S (2.1 g, 8.7 mmol, 3 equiv) was added and the mixture was refluxed for7 h. The THF was evaporated and residues were diluted with 1 M NaOH and extracted with EtOAc (3x50 mL). Layers were separated and organic layers were combined, washed with brine and dried over $MgSO_4$. After evaporation of the solvent, crude product was purified by column chromatography on SiO_2 gradient from DCM to 98/2 DCM/MeOH to give product as an orange powder (0.51 g, 69%). **^{1}H NMR** (601 MHz, DMSO-d6) δ: 10.23 (s, 1H), 7.70 – 7.66 (m, 2H) 7.65 – 7.57 (m, 4H), 6.68 – 6.64 (m, 2H), 5.78 (s, 2H), 2.05 (s, 3H). **HRMS** m/z (ESI): $C_{14}H_{14}N_4O$ $[M+H]^+$,calculated: 255.1246, found: 255.1254.

4. Aniline derivative (**3**) (0.25 g, 0.98 mmol, 1equiv) was added into the mixture of H_2O/EtOH (10/5) and HBF_4 (48% in H_2O, 0.33 mL, 2.6 equiv) at 0˚C. Then cold water (1 mL) solution of $NaNO_2$ (68 mg, 0.98 mmol, 1 equiv) was added over a period of 10 minutes and the mixture was stirred for 1 h at 0˚C. Next, the diazonium salt solution was added dropwise into the solution of 1-naphthylamine (140 mg, 0.98 mmol, 1equiv) and HCl (10 M, 98 µL) at H_2O/EtOH (10/5 mL). The mixture was stirred for 1 h at 0˚C and then left overnight at room temperature. The ethanol was evaporated and mixture basified with ammonium solution and extracted with DCM. Layers were separated and organic layers were combined, washed with brine and dried over $MgSO_4$. After evaporation of the solvent, crude product was purified by column chromatography on SiO_2 gradient from DCM to DCM/MeOH 97/3 to give **4** as a deep purple powder (72 mg, 18%). **^{1}H NMR** (601 MHz, Methanol-d4) δ: 9.02 – 9.00 (m, 1H), 8.11 – 8.03 (m, 5H), 7.99 (d, J = 8.5 Hz, 1H), 7.96 – 7.91 (m, 2H), 7.81 – 7.76 (m, 2H), 7.66 – 7.61 (m, 1H), 7.54 – 7.49 (m, 1H), 6.83 (d, J = 8.5 Hz, 1H), 2.18 (s, 3H). ^{13}C NMR (151 MHz, Methanol-d4) δ: 171.9, 156.4, 154.0, 151.8, 150.3, 143.1, 140.1, 135.3, 128.5, 125.9, 124.9, 124.7, 124.4, 123.3, 123.0, 121.1, 116.3, 109.2. **HRMS** m/z (ESI): $C_{24}H_{20}N_6O$ $[M+H]^+$,calculated: 409.1777, found: 409.1768.

3c: To a solution of **4** (30 mg, 7.3 mmol, 1 equiv) in methanol (6 mL), 1 mL of 6 M HCl was added and heated at 75°C for 8 h. Methanol was evaporated and the mixture was basified with 1 M NaOH and extracted with DCM (3x20 mL). Layers were separated and organic layers were combined, washed with brine and dried over $MgSO_4$. After evaporation of the solvent, crude product was purified by column chromatography on SiO_2 gradient from DCM to DCM/MeOH 98/2 to give product as a deep purple powder (20 mg, 75%). **^{1}H NMR** (400 MHz, Methylene Chloride-d2) δ: 9.09 (d, J = 7.9 Hz, 1H), 8.12 – 8.06 (m, 2H), 8.03 – 7.96 (m, 3H), 7.89 – 7.81 (m, 3H), 7.73 – 7.65 (m, 1H), 7.62 – 7.55 (m, 1H), 6.86 (d, J = 8.4 Hz, 1H), 6.80 – 6.75 (m, 2H), 4.81 (s, 1H), 4.22 (s, 1H). **^{13}C NMR** (101 MHz, Methylene Chloride-d2) δ 154.1, 153.4, 150.4, 147.2, 145.6, 140.3, 133.4, 127.4, 125.4, 125.3, 123.9, 123.4, 123.2, 122.3, 120.8, 114.5, 114.2, 109.0. **HRMS** m/z (ESI): $C_{22}H_{18}N_6$ $[M+H]^+$,calculated: 367.1671, found: 367.1667.

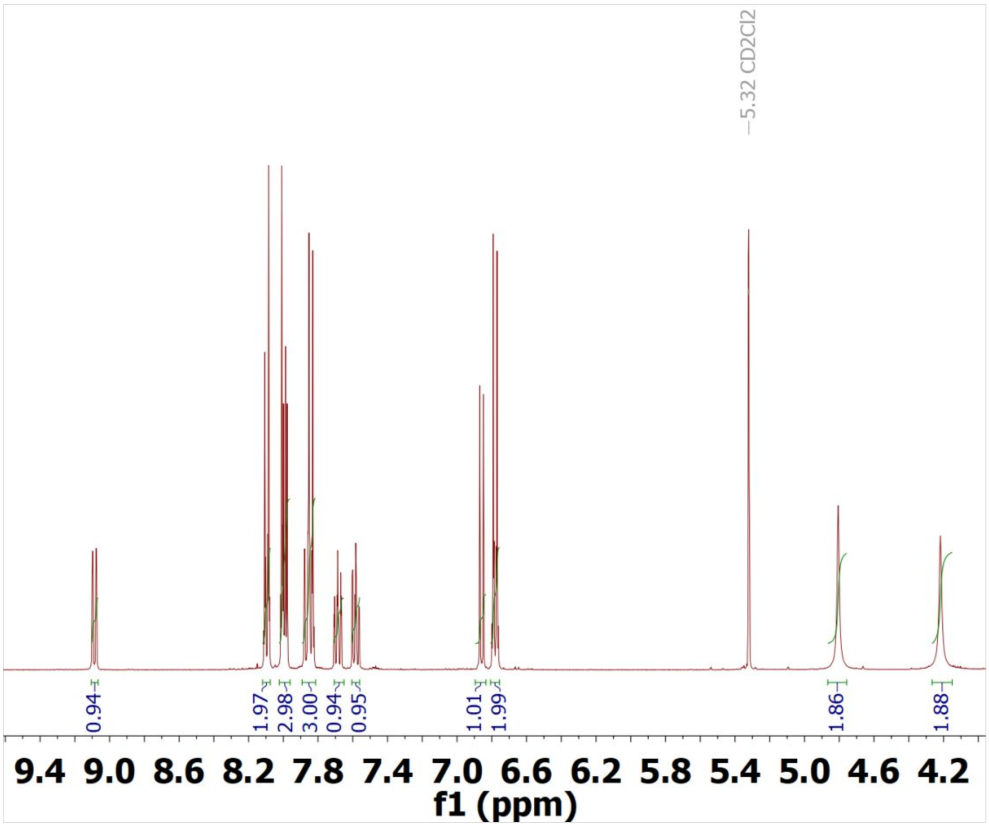

Figure S12. ^{1}H NMR spectrum of **3c**.

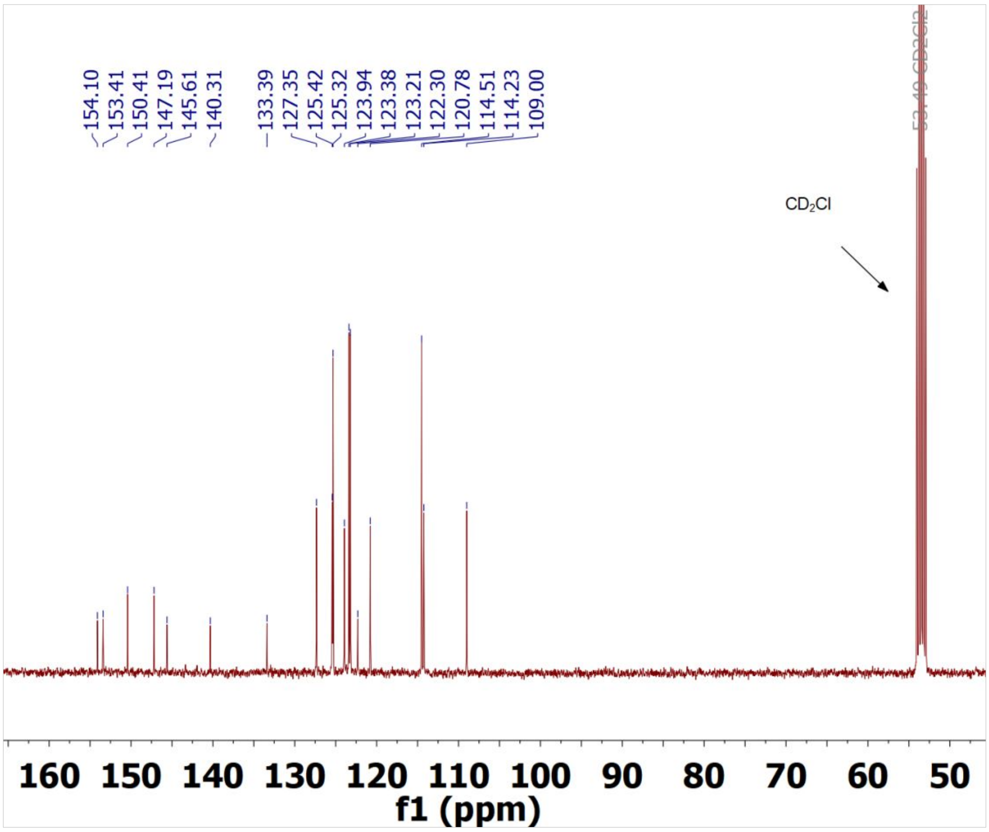

Figure S13. ^{13}C NMR spectrum of **3c**.

3. PHOTOISOMERIZATION STUDIES

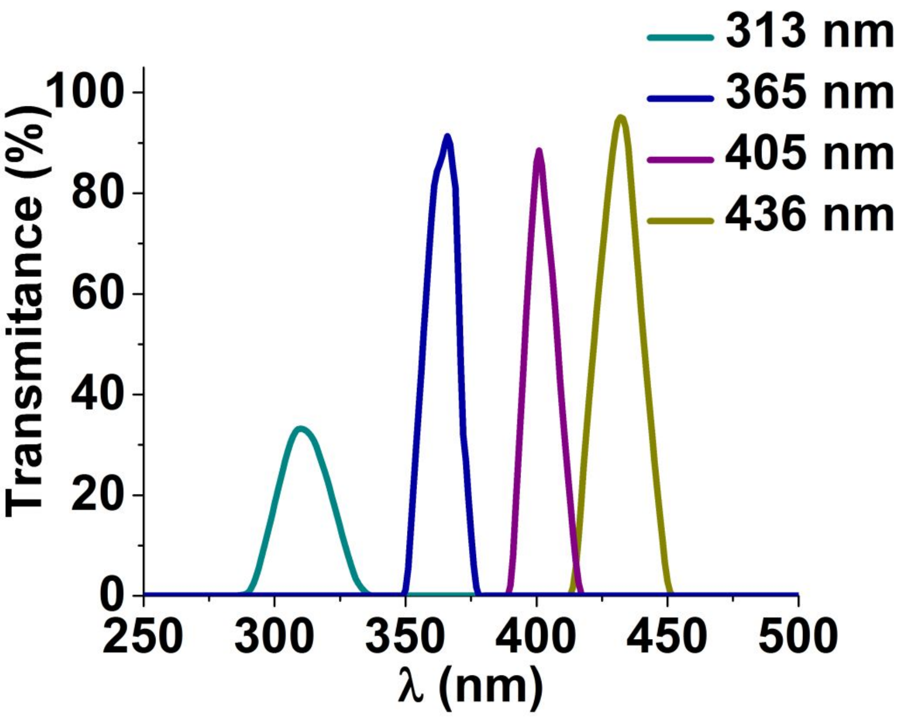

Figure S14. Transmittance spectra of the filters used for irradiation.

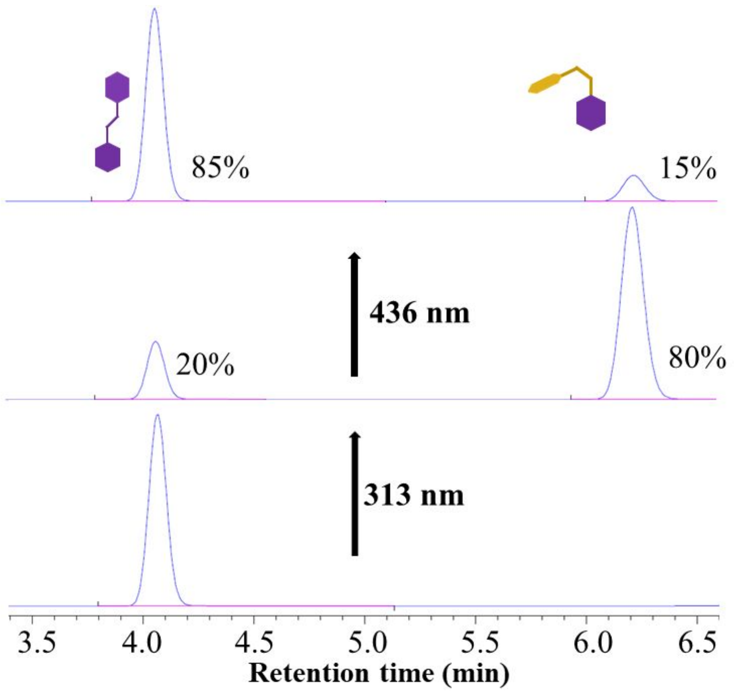

Figure S15. Chromatograms of **1a** after irradiation with 313 nm and then 436 nm, the composition of $PSS_{313\ nm}$ found to be 80/20% of *Z/E* and that at $PSS_{436\ nm}$ found to be 15/85% *Z/E*.

4. THERMAL STABILITY

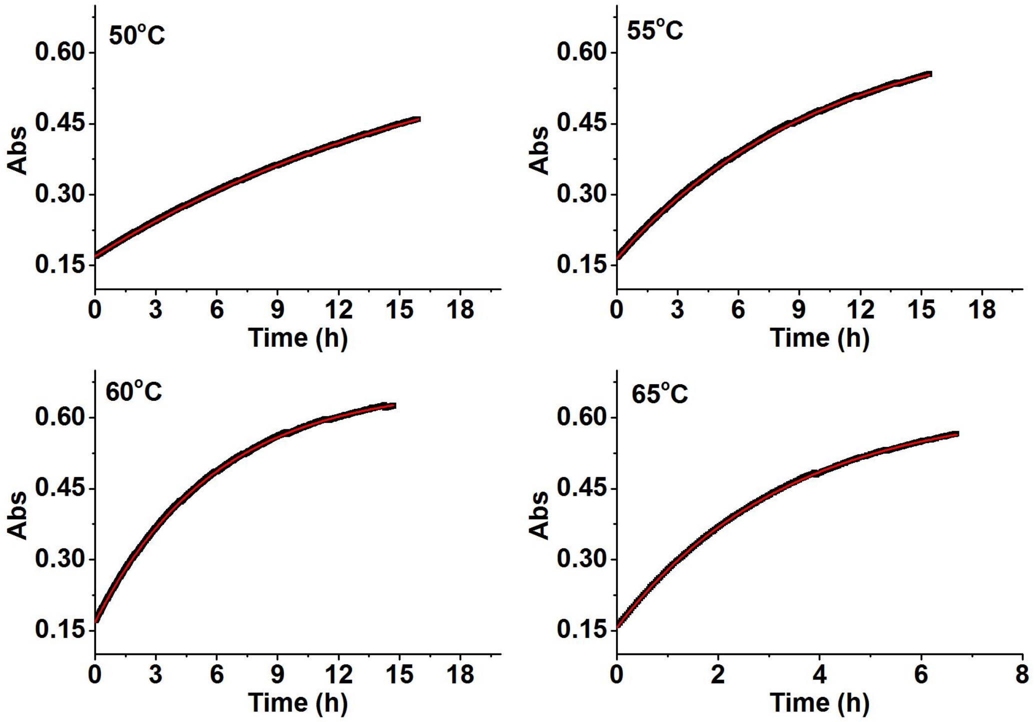

Figure S16. Thermal **1a**-*Z* to **1a**-*E* isomerization in acetonitrile at four temperatures 50˚C, 55˚C, 60˚C, 65˚C. Red lines represent the data fitting curve (first-order kinetics).

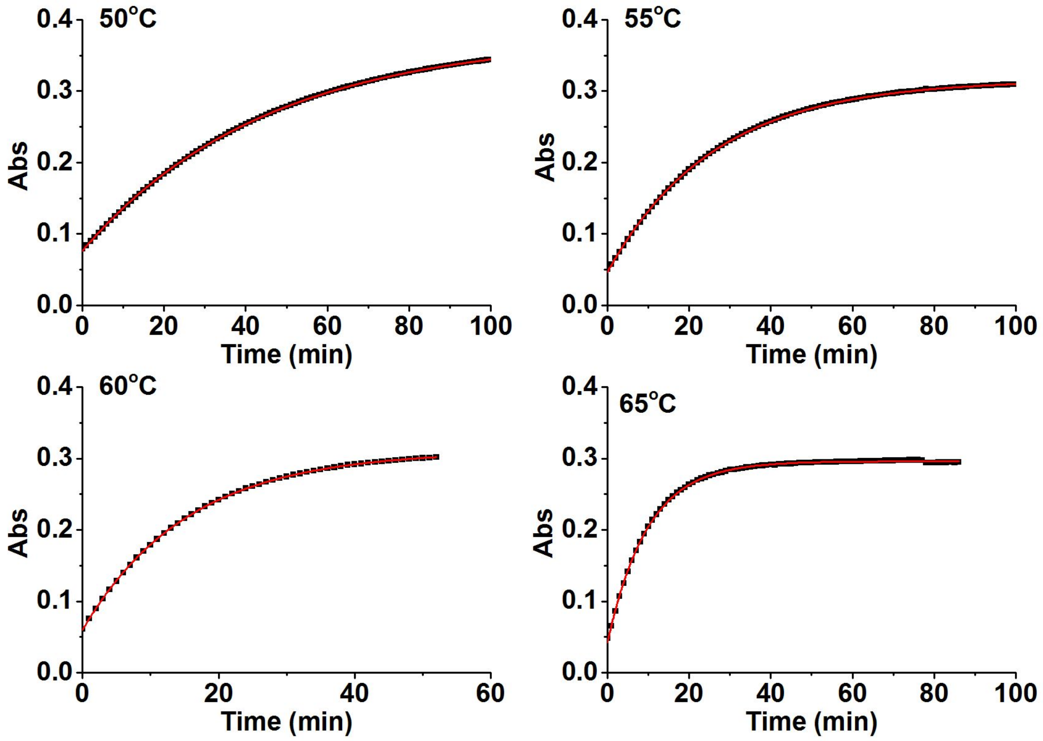

Figure S17. Thermal **2a**-*Z* to **2a**-*E* isomerization in acetonitrile at four temperatures 50˚C, 55˚C, 60˚C, 65˚C. Red lines represent the data fitting curve (first-order kinetics).

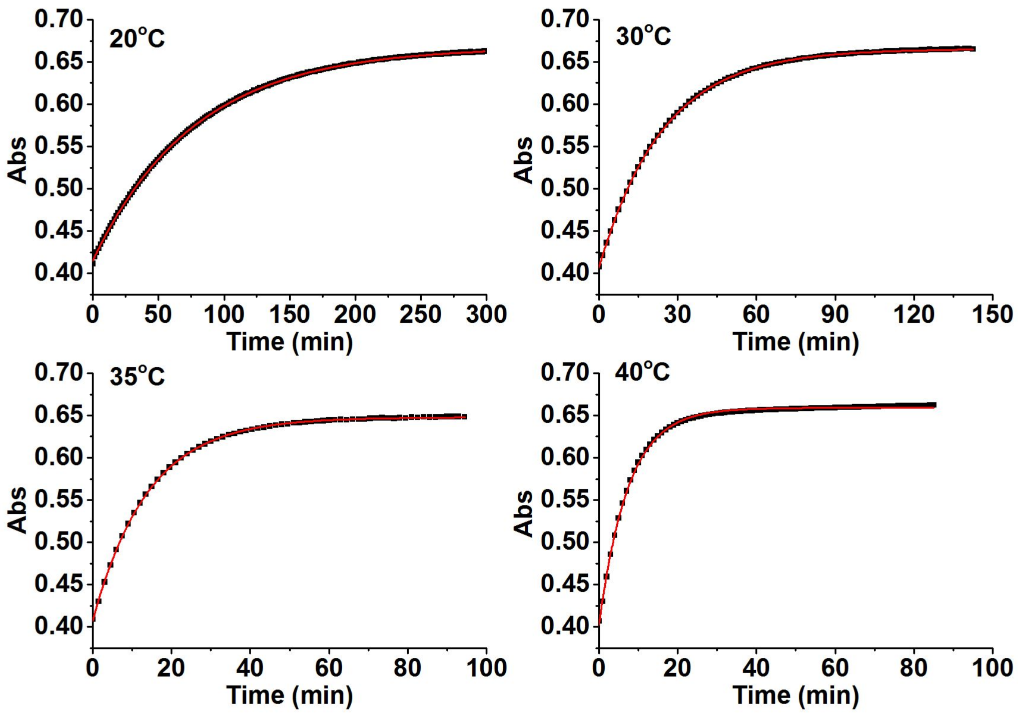

Figure S18. Thermal **3a-***Z* to **3a-***E* isomerization in acetonitrile at four temperatures 20°C, 30°C, 35°C, 40°C. Red lines represent the data fitting curve (first-order kinetics).

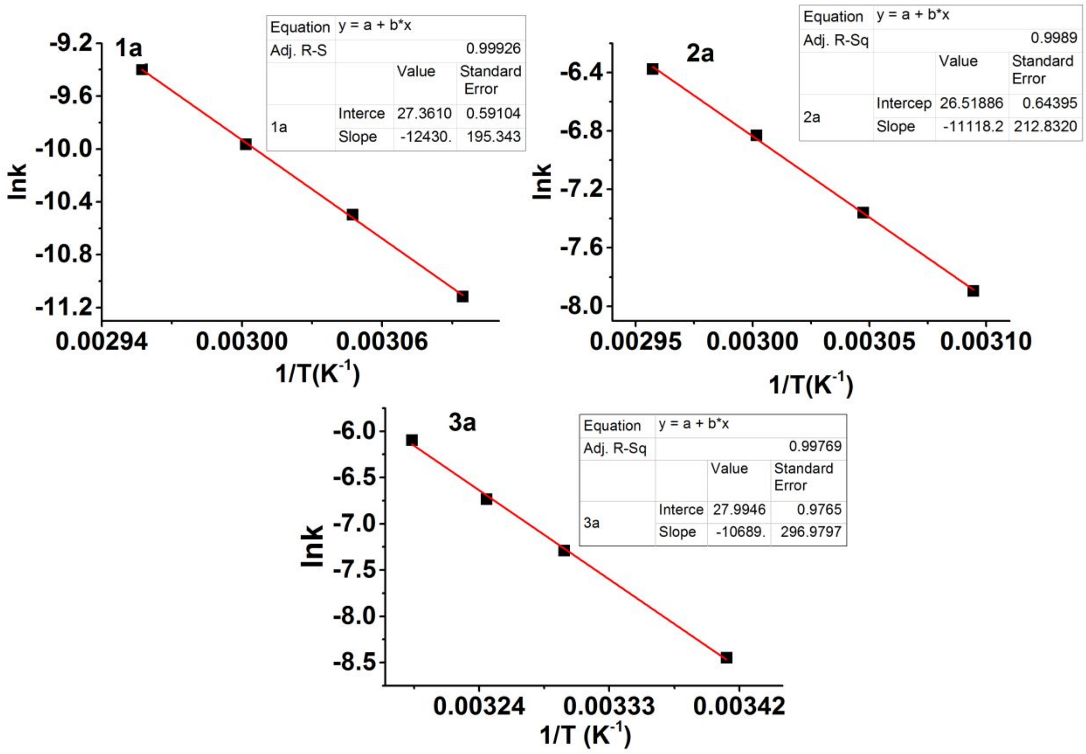

Figure S19. Arrhenius plots for **1a**, **2a** and **3a** in acetonitrile, rate constants of the *Z-E* isomerization process at different temperatures were determined by the exponential curve fitting of the plot: absorbance vs. time. The slope is equal to $-E_a/R$.

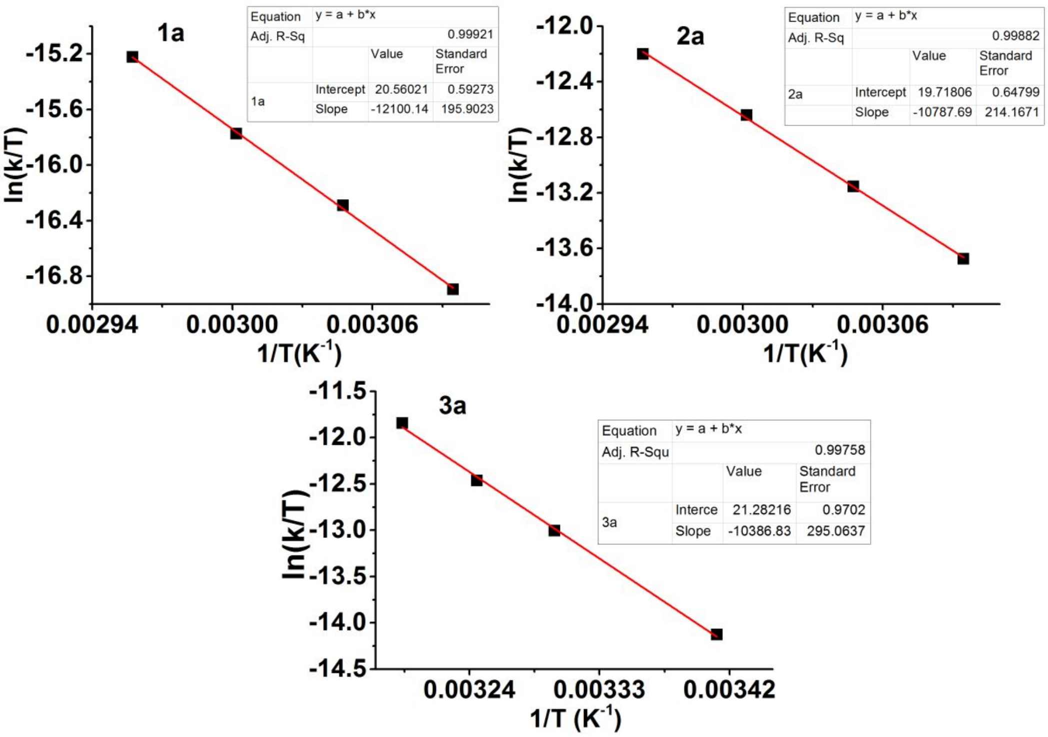

Figure S20. Eyring plots for **1a**, **2a** and **3a** in acetonitrile, the slope is equal to -ΔH‡/R, intercept: $\ln((\kappa k_B)/h) + \Delta S^‡/R$.

Table S1. Isomerization rates $k_{Z\rightarrow E}$ (at 298 K), thermal half-life $\tau_{1/2}$ (at 298 K), Arrhenius prefactor A, activation energies Ea, as well as Eyring activation free energies ΔG‡, enthalpies ΔH‡ and entropies ΔS‡ for compounds **1a**, **2a** and **3a** in acetonitrile.

**Cmpd**	$k^{[a]}_{E\rightarrow Z}$ [s^{-1}]	$\ln k_{E\rightarrow Z}$	$\tau_{1/2}$ [h]	A[c] [s^{-1}]	**Ea** $[\frac{kJ}{mol}]$	**ΔH‡** $[\frac{kJ}{mol}]$	**ΔS‡** $[\frac{J}{mol\times K}]$	**ΔG‡** $[\frac{kJ}{mol}]$
**1a**	6.0 ± 2.9	-14.3 ± 0.7	322 ± 155	7.6 ± 3.4	103.3 ± 1.6	100.6 ± 1.6	-26.6 ± 4.9	108.5 ± 2.2
**2a**	209 ± 110	-10.8 ± 0.7	9.2 ± 4.8	3.3 ± 1.6	92.4 ± 1.8	89.7 ± 1.8	-33.6 ± 5.4	99.7 ± 2.4
**3a**	3760 ± 2400	-7.9 ± 1.0	0.51 ± 0.30	14.4 ± 8.9	88.9 ± 2.5	86.3 ± 2.5	-20.6 ± 8.1	92.5 ± 3.5

[a] 10^{-7} at 298 K. [b] estimated from Arrhenius equation at 298 K. [c] 10^{11}

5. Z-SCAN STUDIES

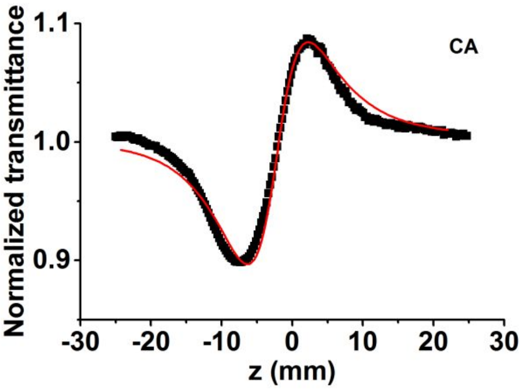

Figure S21. Representative Z-scan traces of **1c** at 750 nm: left closed-aperture (CA) (black squares); right open-aperture (OA) (black squares). The red curves correspond to the theoretical curves.

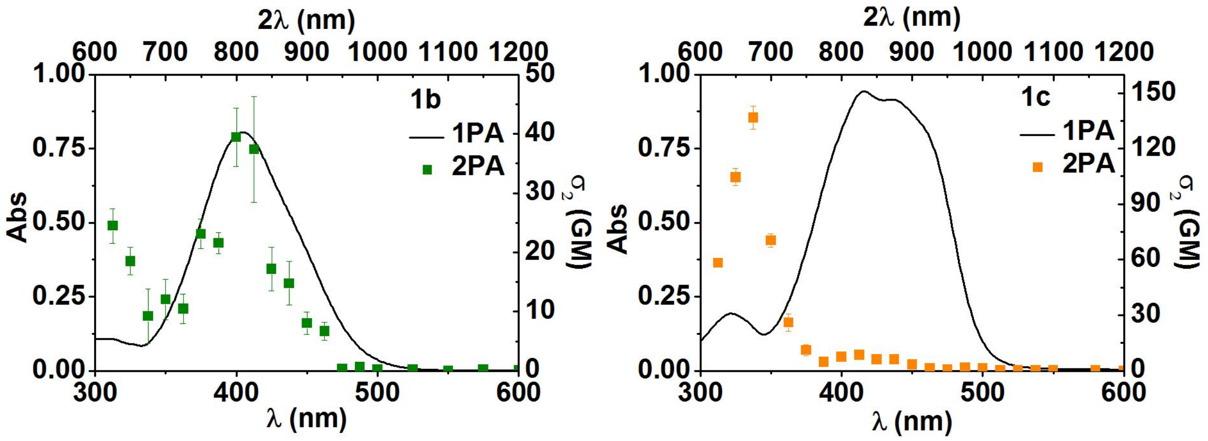

Figure S22. Overlay of one and two-photon absorption spectra for **1b** (left) and **1c** (right) in DMSO at 20 °C.

6. THEORETICAL CALCULATIONS

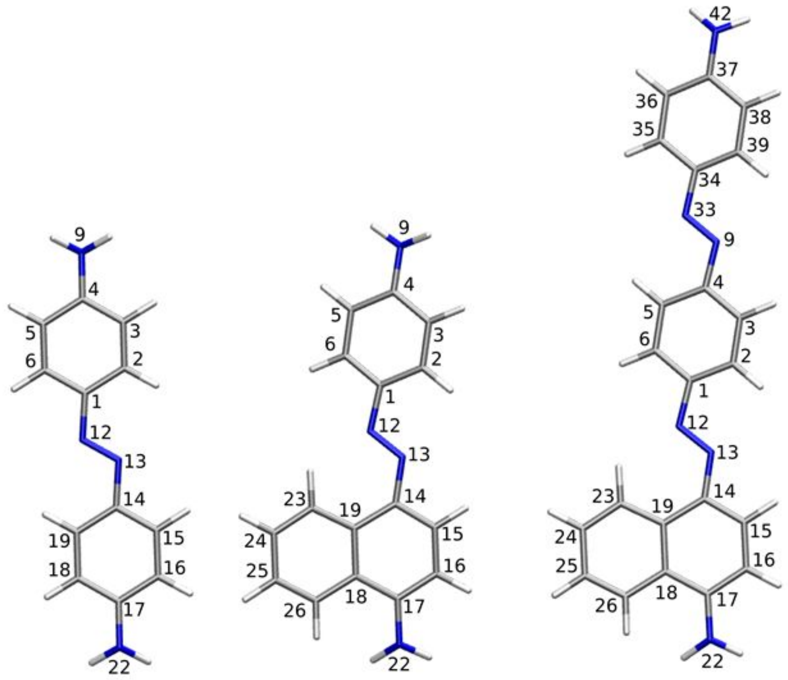

Figure S23. Atom numbering.

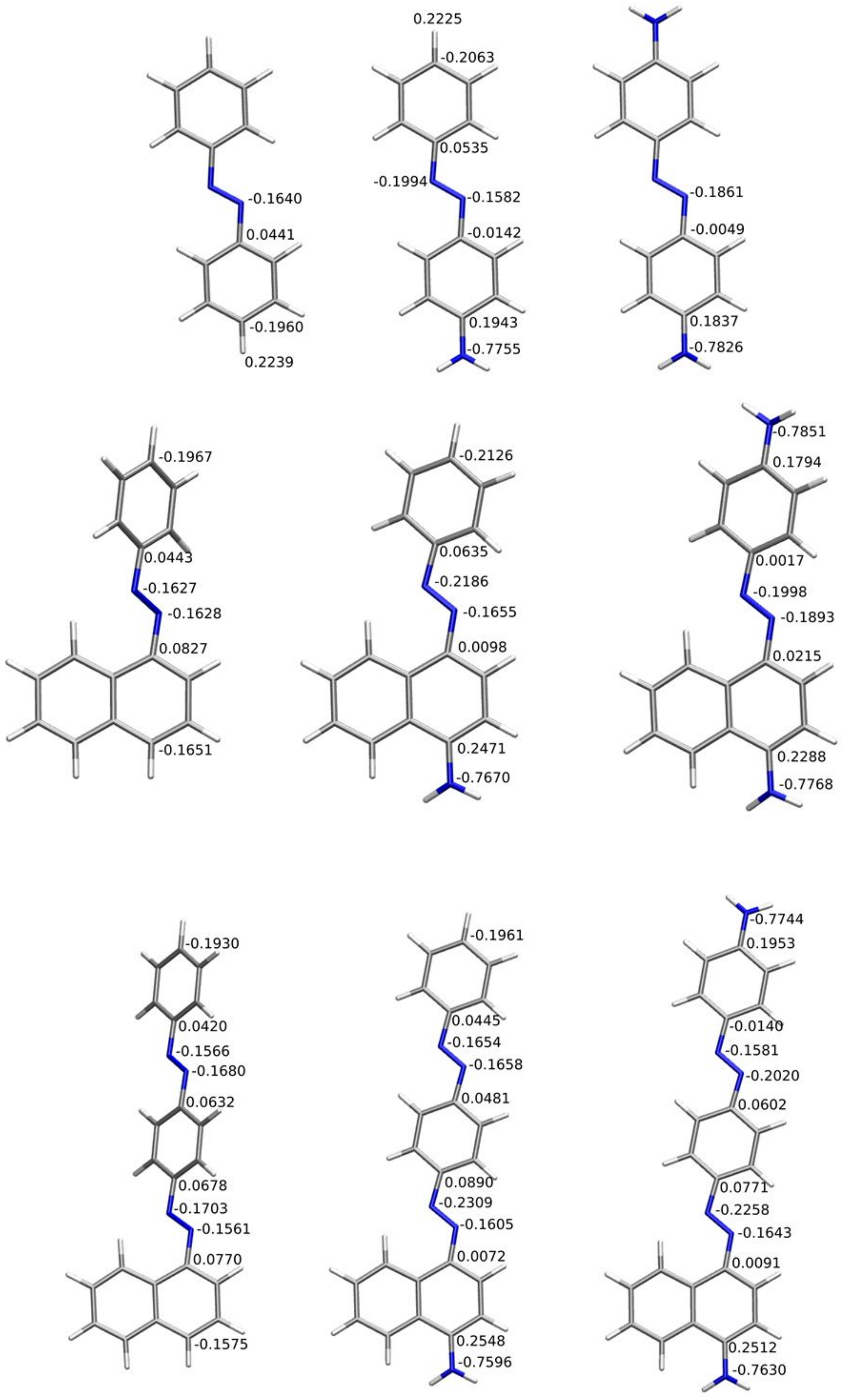

Figure S24. NBO partial atomic charges estimated within the ωB97X-D/def2-TZVP/PCM(DMSO) approach.

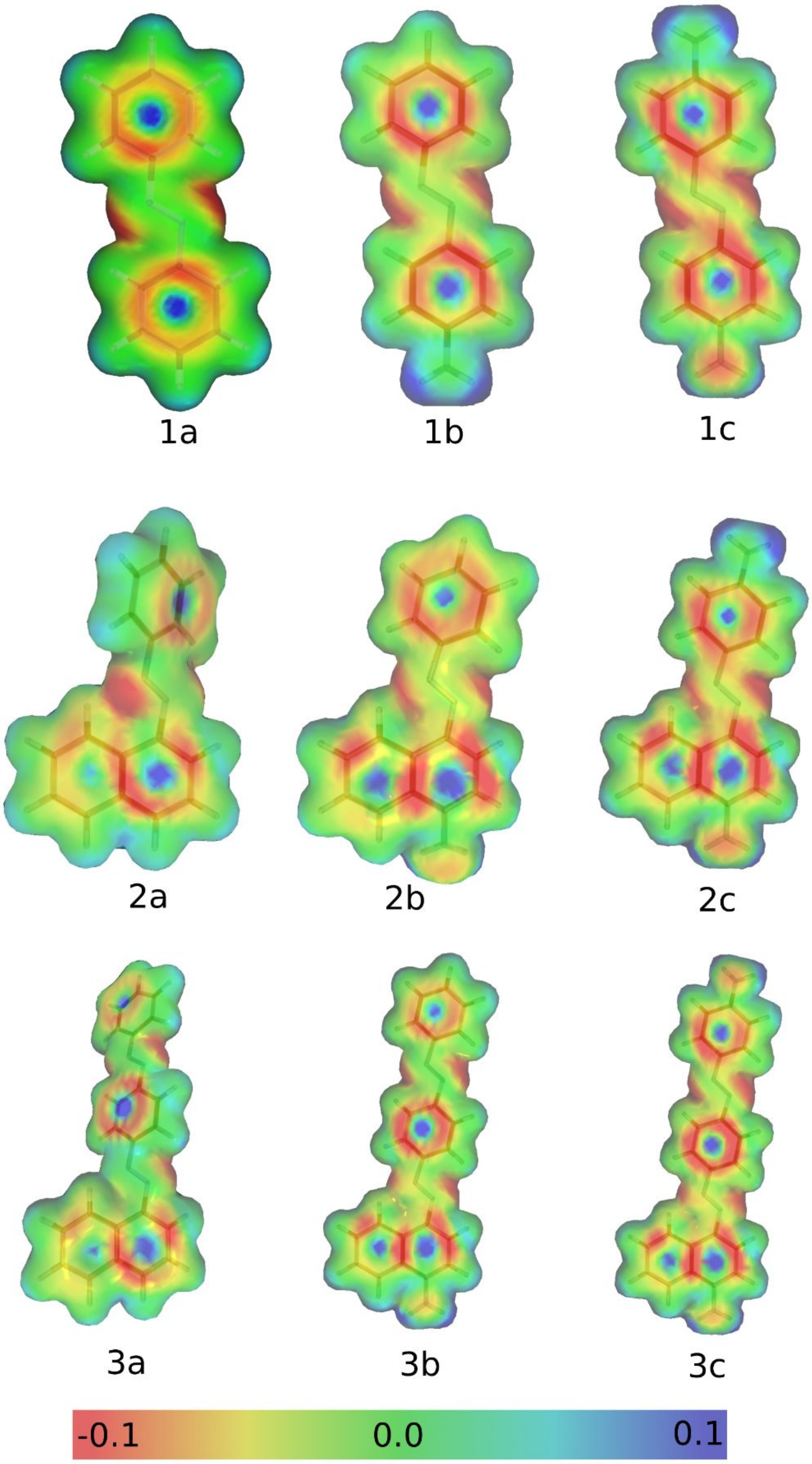

Figure S25. Electrostatic potential for **1a**-**3c** for optimized structures.

The pyramidalization of the amino group is a natural feature of the amino-compounds, explained by their tendency to adopting of the sp^3 hybridization at nitrogen. The pyramidalization angle for the amino groups in the analyzed systems is presented in the table below with the dihedral angles H-N-C-H as its measure (-180 degrees would correspond to the planar $-NH_2$ group):

Table S2. The pyramidalization angle for the amino groups of the investigated compounds.

	**wB97X-D/def2-TZVP/PCM(DCM)**	
Dihedral angle [degrees]:	H24-N9-C4-H25	H27-N22-C17-H28
1b-*E*	-142.06	
1c- *E*	-138.60	-138.60
2b- *E*	-142.46	
2c- *E*	-137.96	-137.40
3b- *E*	-145.95	
3c- *E*	-144.30	-142.48

It can be clearly noticed that the amino-group is not planar, the nitrogen is slightly (about 3 degrees) pushed below the surface, but the hydrogens significantly deviate from planarity (C-C-N-H dihedrals all about 20 degrees). Thus, it will break the planar symmetry of the C_{2h} point group observed for the ideal unsubstituted azobenzene **1a**, and in the case of 1c lead to C_i-like symmetry, with the pyramidalization of both groups in opposite directions. The growing size of the analyzed azobenzene molecules additionally results in the asymmetric pyramidalization of both amino groups present in the system. Namely, for **1c** both amino groups are characterized with the same dihedral angle (Table S2) equal to -138.6 degrees. For **2c**, the small difference of these two dihedrals appears and it gets more pronounced for **3c** (-144 .30 versus -142.48 degrees). This introduces further deviation from the C_{2h}-like symmetry point group.

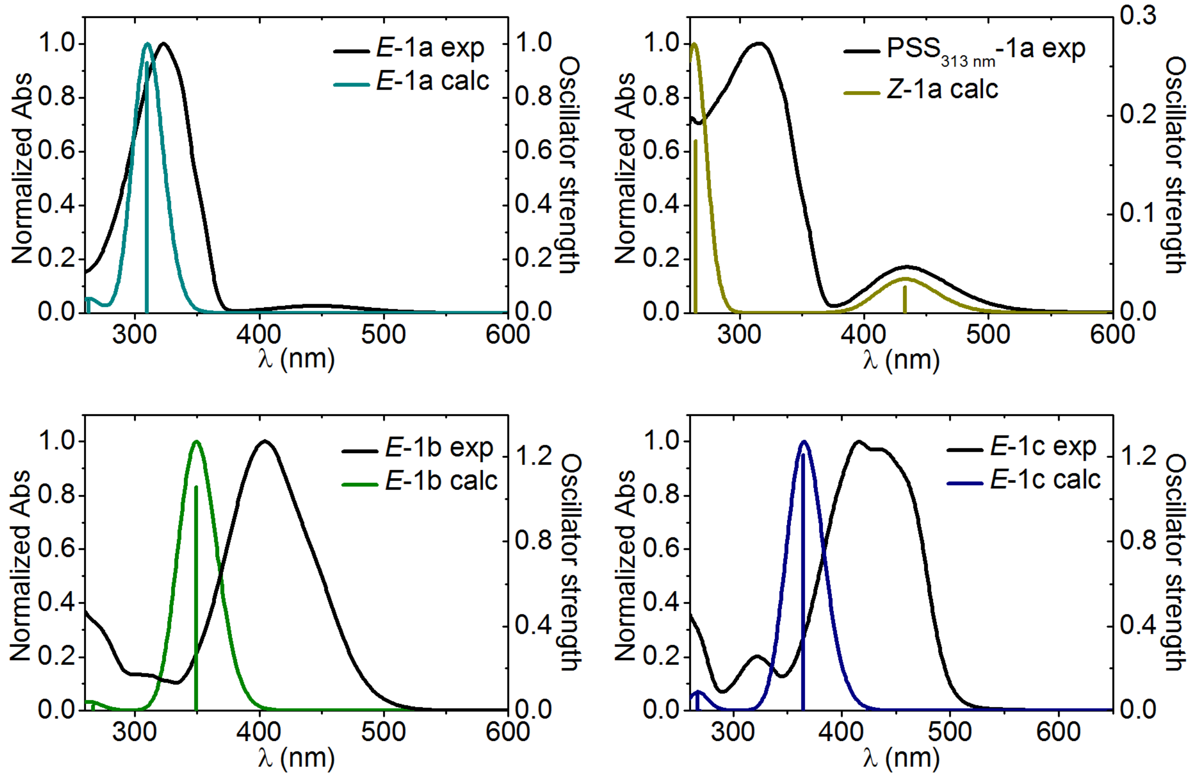

Figure S26. Normalized experimental and calculated UV-Vis spectra of **1a-1c** in DMSO.

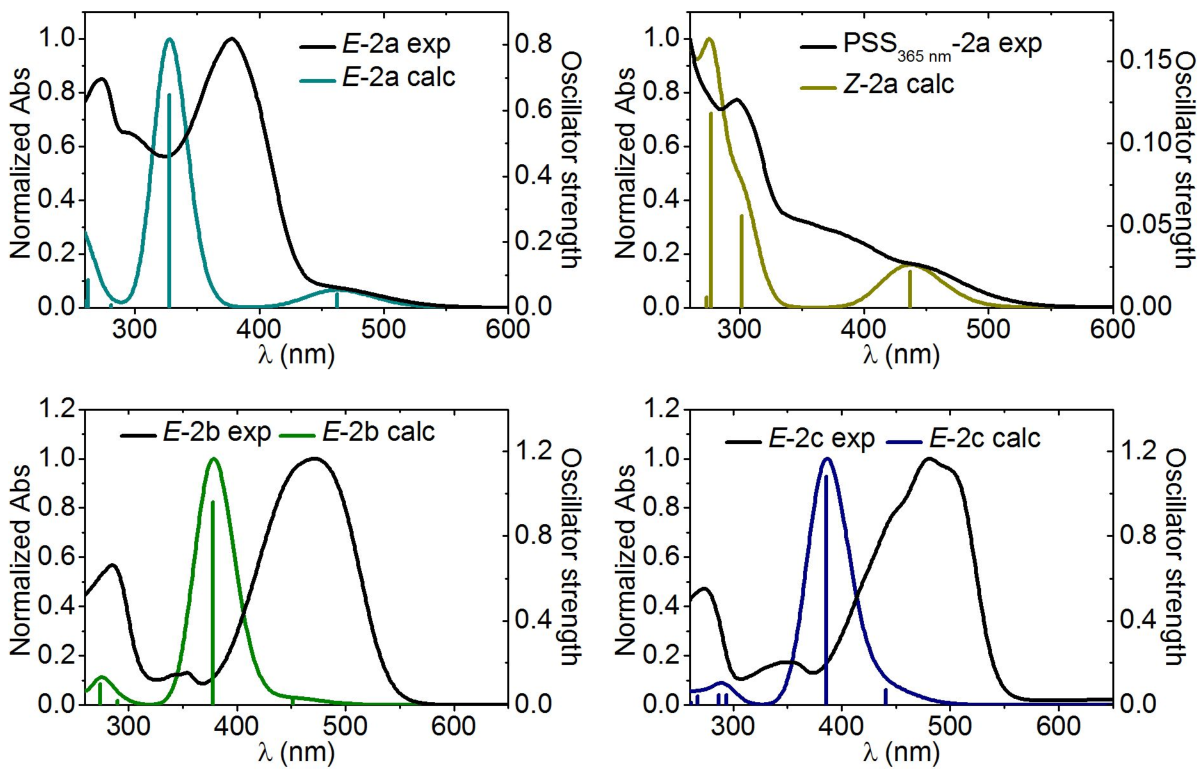

Figure S27. Normalized experimental and calculated UV-Vis spectra of **2a-2c** in DMSO.

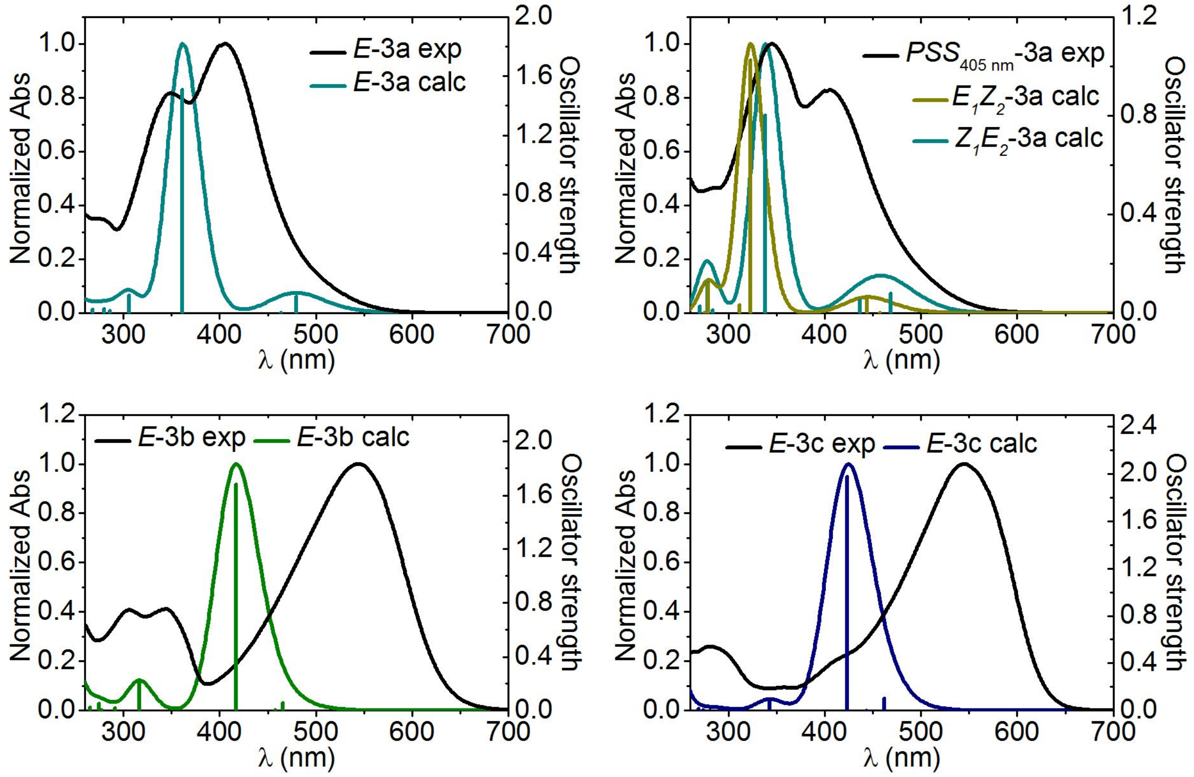

Figure S28. Normalized experimental and calculated UV-Vis spectra of **3a-3c** in DMSO.

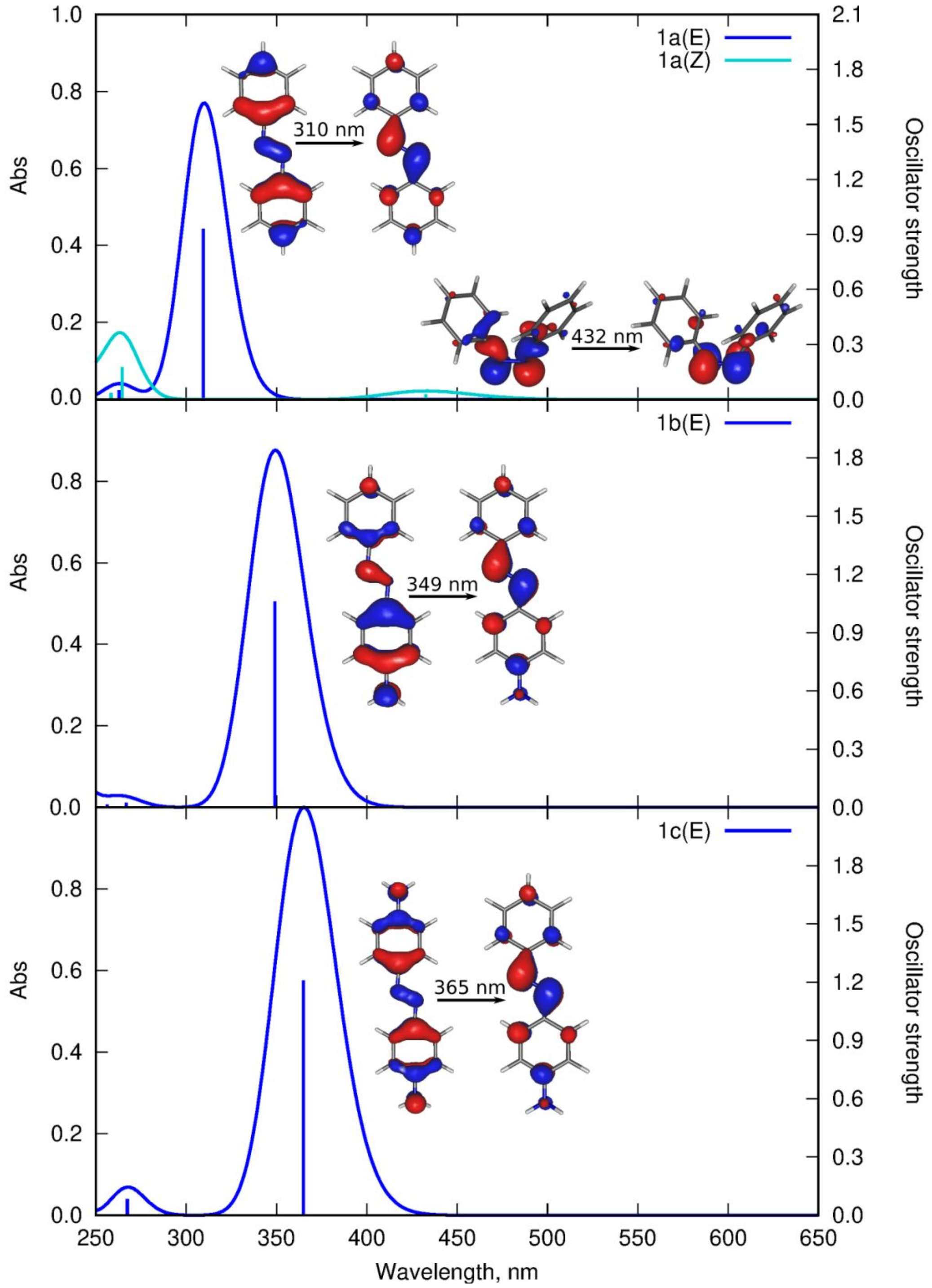

Figure S29. Vertical absorption spectrum estimated in B97X-D/def2/TZVP/PCM(DMSO) approach together with the most important natural transition orbitals for **1a**, **1b** and **1c** systems.

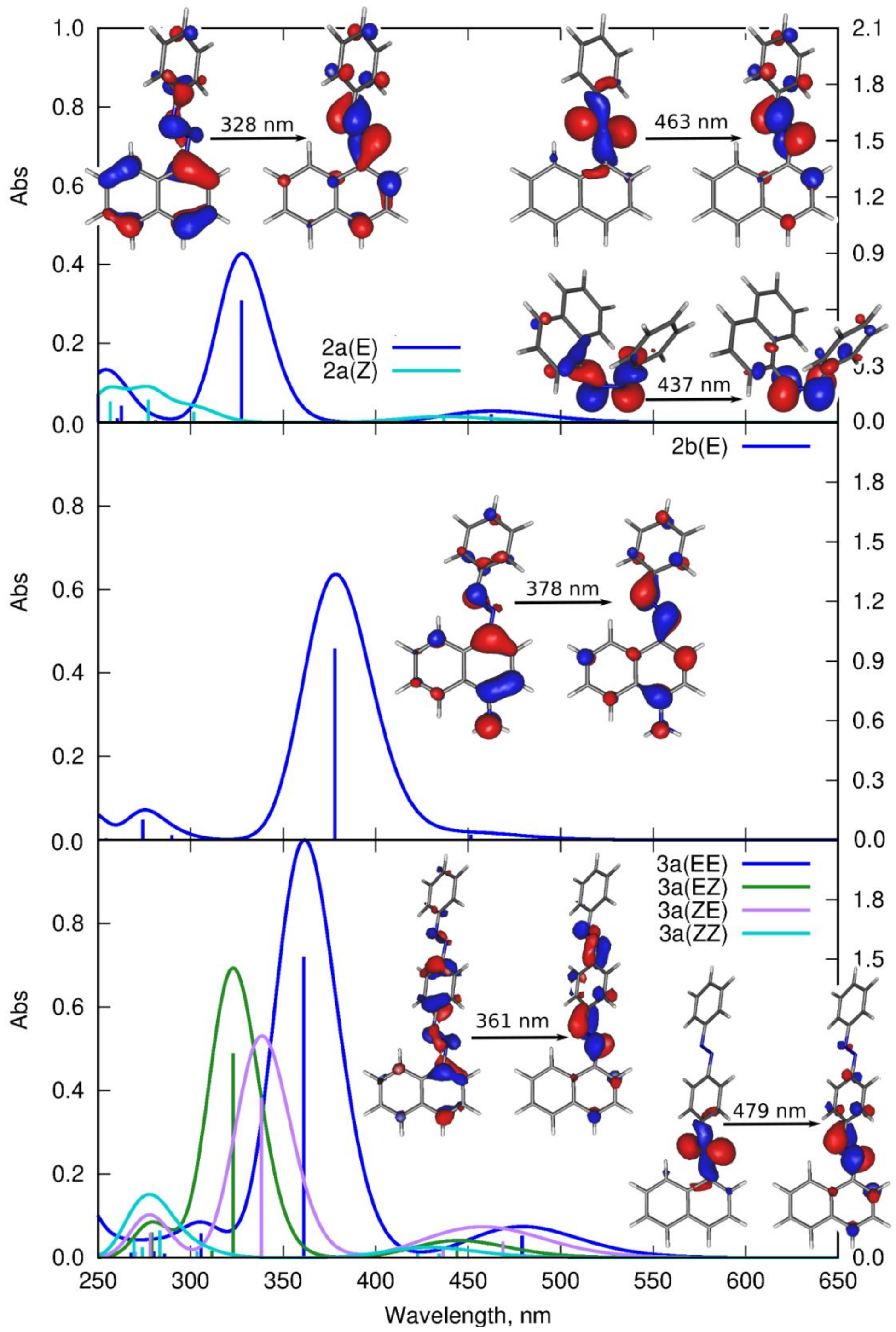

Figure S30. Vertical absorption spectrum estimated in ωB97X-D/def2/TZVP/PCM(DMSO) approach together with the most important natural transition orbitals for **2a**, **2b** and **3a** systems.

Table S3. Second-order perturbation theory analysis of the Fock matrices in NBO basis for systems containing one and two amino groups: LP denotes the lone pair at the corresponding nitrogen of the NH_2 group, the BD* stands for the antibonding orbital in the aromatic ring. E(2) describes the stabilization arising from the donor-acceptor interaction, E(j) and E(i) are the acceptor and donor orbital energies, respectively and F(i,j) are the resonance integrals of the real-world one electron effective Hamiltonian. Data presented for E isomer.

	Donor NBO (i)	Acceptor NBO (j)	E(2) (kcal/mol)	E(j)-E(i) (a.u.)	F(i,j) (a.u.)
			NH_2 at the phenyl/naphthalene ring		
**1b**	LP N9	BD* C4-C5	48.37	0.42	0.134
**2b**	LP N22	BD* C16-C17	53.48	0.43	0.140
**3b**	LP N22	BD* C16-C17	57.83	0.42	0.144
			NH_2 at the terminal phenyl ring		
**1c**	LP N9	BD* C4-C5	44.39	0.43	0.131
**2c**	LP N9	BD* C4-C5	43.01	0.43	0.129
**3c**	LP N42	BD* C36-C37	48.97	0.42	0.135
			NH_2 at the phenyl/naphthalene ring		
**1c**	LP N22	BD* C16-C17	44.39	0.43	0.131
**2c**	LP N22	BD* C16-C17	47.30	0.44	0.134
**3c**	LP N22	BD* C16-C17	55.75	0.42	0.142

Table S4. Relative energy, relative Gibbs free energy and the molar fraction of the Z_1Z_2, Z_1E_2, E_1Z_2 and E_1E_2 isomers of **3a** in the mixture in standard conditions (ωB97X-D/def2/TZVP/PCM(DMSO))

	Relative energy (kcal/mol)	Relative Gibbs free energy, (kcal/mol)	Molar fraction
Z_1Z_2	15.41	18.18	0.9999
Z_1E_2	9.86	11.20	0.0000
E_1Z_2	7.13	8.54	0.0000
E_1E_2	0.00	0.00	0.0000

Table S5. Ground (GS) and excited (ES) states dipole moments calculated using ωB97X-D/def2-TZVP/PCM(DMSO) level of theory.

Compound		μ^{GS} (D)	λ_{1PA} (nm)	μ^{ES} (D)
**1a**	***E***	0.00	310<br>454	0.00<br>0.00
	Z	4.27	433	3.83
**1b**		3.95	349	10.25
**1c**		0.00	365	0.00
**2a**	***E***	0.23	328	4.35
	Z	4.37	437	4.31
**2b**		5.01	378	9.33
**2c**		0.80	386<br>441	1.04
**3a**	E_1E_2	0.28	361<br>479	2.83<br>3.06
	E_1Z_2	4.25	323	4.60
	Z_1E_2	4.51	338	7.22
	Z_1Z_2	4.62	283	3.79
**3b**		6.11	417<br>466	7.32<br>13.08
**3c**		2.49	424<br>462	3.27<br>4.57

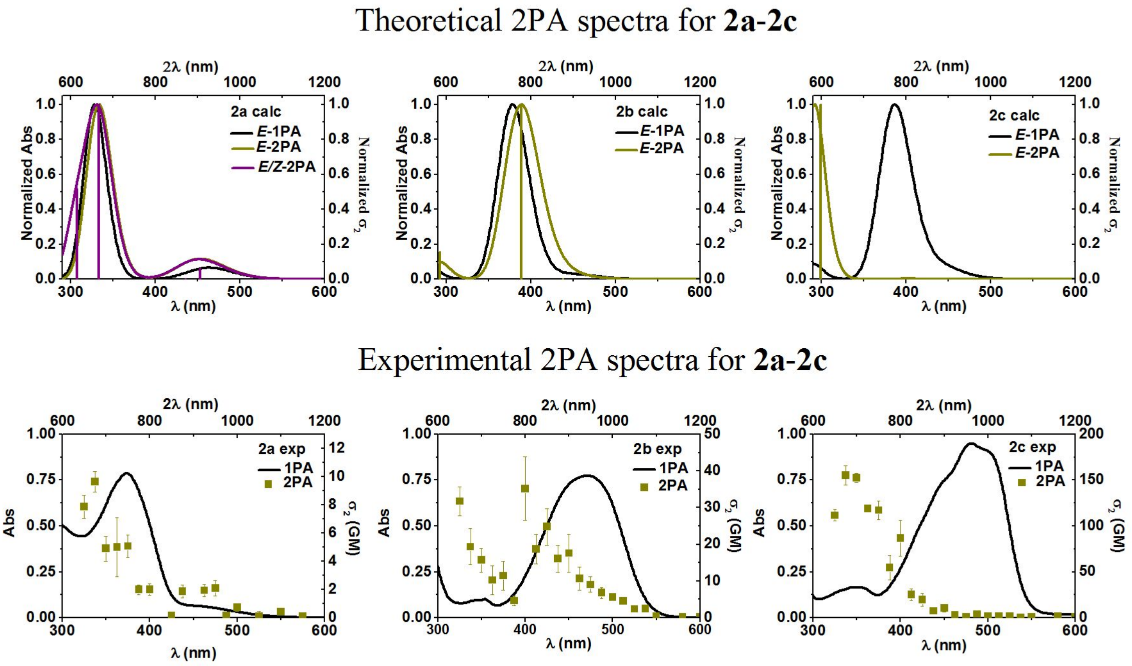

Figure S31. Overlay of 1PA and 2PA for **2a-c**, calculated with the CAM-B3LYP/def2-TZVP/PCM(DMSO) (upper panel) and experimentally found (lower panel) in DMSO at 20 °C.

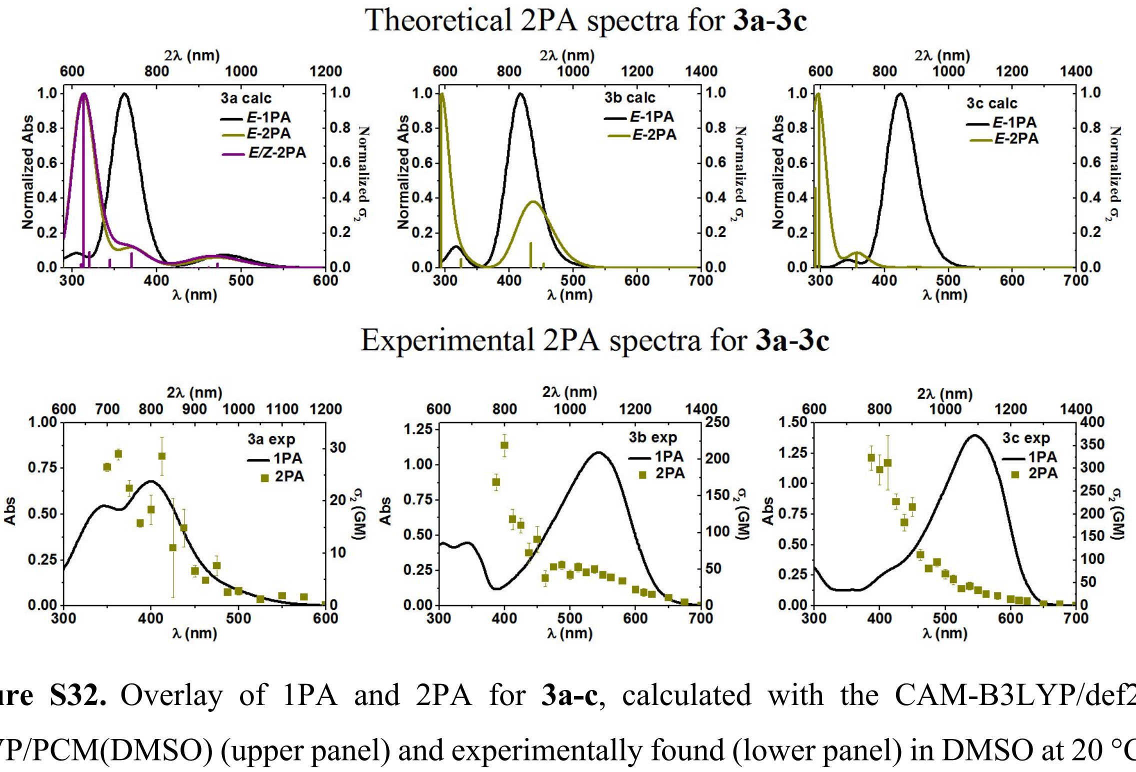

Figure S32. Overlay of 1PA and 2PA for **3a-c**, calculated with the CAM-B3LYP/def2-TZVP/PCM(DMSO) (upper panel) and experimentally found (lower panel) in DMSO at 20 °C.

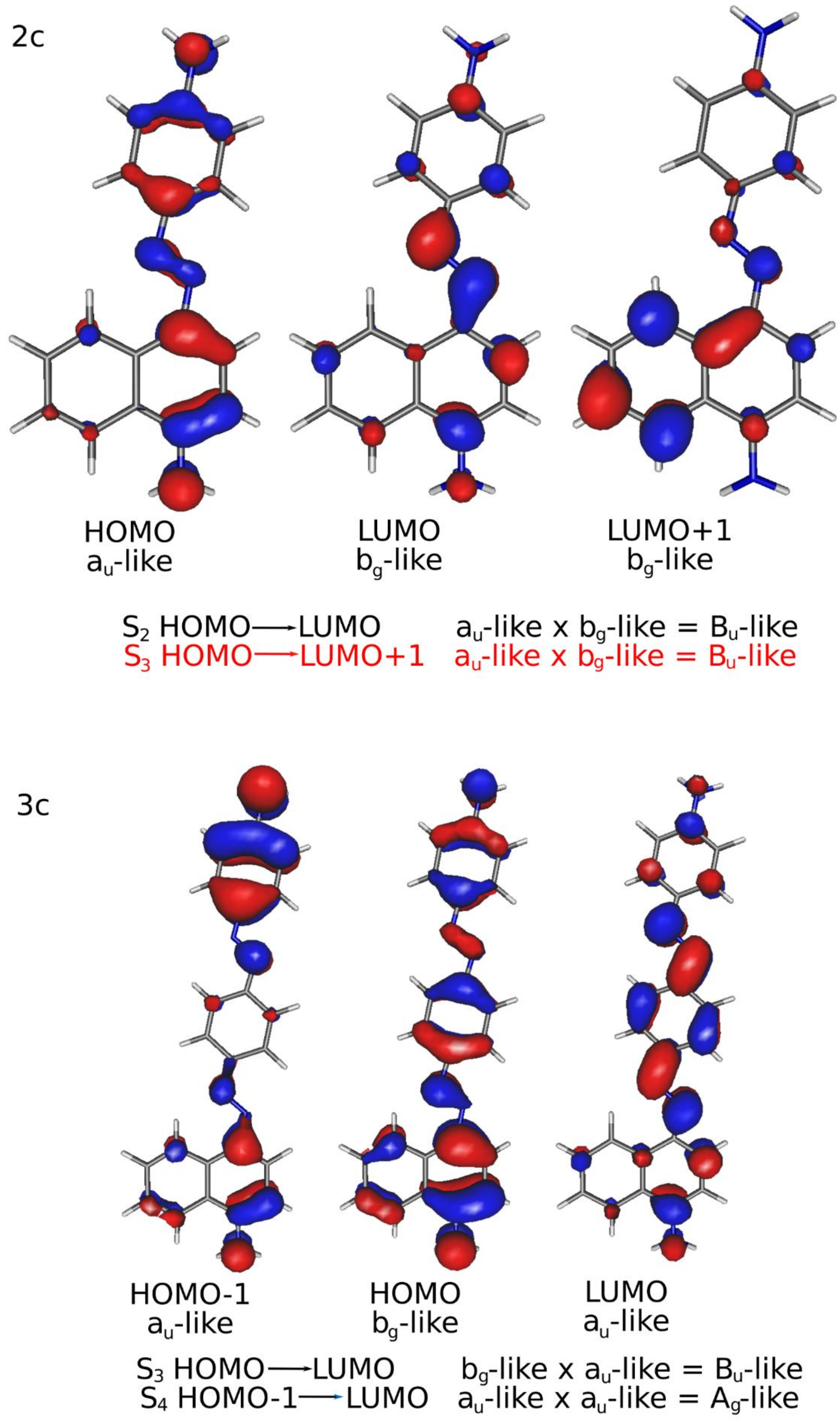

Figure S33. Frontier molecular orbitals involved in the most intensive 1PA and 2PA transitions and their symmetry for the C_{2h}-like point group.

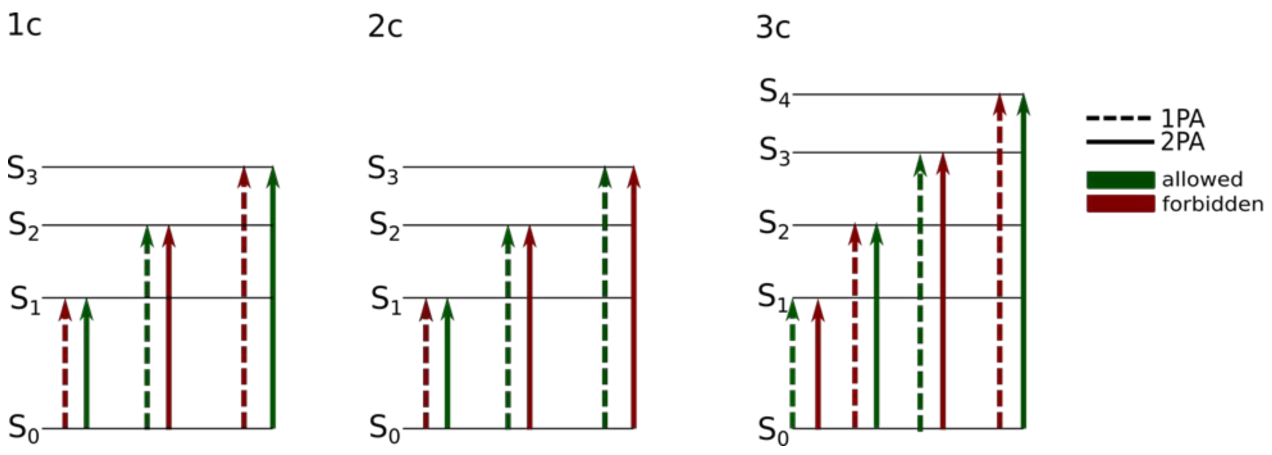

Figure S34. Schematic presentation of the states for **1c**, **2c** and **3c** with 1PA and 2PA symmetry-allowed and symmetry-forbidden transitions.

Table S6. Symmetry assignment for the frontier orbitals in the C_{2h}-like point group for **2c** and **3c**

**2c orbital**	E	C_2	i	σ_h	Representation
HOMO	1	1	-1	-1	a_u-like
LUMO	1	-1	1	-1	b_g-like
LUMO+1	1	-1	1	-1	b_g-like
**3c orbital**	E	C_2	i	σ_h	Representation
HOMO-1	1	1	-1	-1	a_u-like
HOMO	1	-1	1	-1	b_g-like
LUMO	1	1	-1	-1	a_u-like

Table S7. Oscillator strength corresponding to the intensity of the 1PA signals, 2PA cross sections, transition character, numbers of involved frontier orbitals and symmetry of the transition for 1c(E), 2c(E) and 3c(EE); last two columns contain the indication of the forbidden (F) or allowed (A) transition according to the parity considerations based on Laporte rule

	1PA	2PA	1PA	2PA	1PA	2PA				1PA	2PA
	**1a(E)**		**1b(E)**		**1c(E)**						
S0→S1	0.00	0	0.00	0	0.00	0	n-> π^*	54->57	a_g x $b_g=B_g$	F	A
S0→S2	0.92	0	1.05	115	1.20	0	π-> π^*	56->57	a_u x $b_g=B_u$	A	F
S0→S3	0.05	0	0.02	4	0.00	1946	π-> π^*	56->59	a_u x $a_u=A_g$	F	A
	**2a(E)**		**2b(E)**		**2c(E)**						
S0→S1	0.05	2	0.03	1	0.10	0	n-> π^*	67->70	a_g x $b_g=B_g$	F	A
S0→S2	0.62	33	0.95	59	1.05	1	π-> π^*	69->70	a_u x $b_g=B_u$	A	F
S0→S3	0.01	7	0.02	10	0.05	125	π-> π^*	69->71	a_u x $b_g=B_u$	A	F
	**3a(EE)**		**3b(EE)**		**3c(EE)**						
S0→S1	0.14	16	0.11	76	0.24	18	n-> π^*	94->97	a_g x $a_u=A_u$	A	F
S0→S2	0.00	0	0.01	10	0.01	16	n-> π^*	95->97	a_u x $a_u=A_g$	F	A
S0→S3	1.45	51	1.60	1.60	1.82	0	π-> π^*	96->97	b_g x $a_u=B_u$	A	F
S0→S4	0.14	607	0.15	0.15	0.07	1651	π-> π^*	95->97	a_u x $a_u=A_g$	F	A

7. REFERENCES

(1) Zhang, Y.; Xue, P.; Yao, B.; Sun, J. Self-assembly of azobenzene-based two-component gels. *New J. Chem.* **2014**, *38*, 5747-5753.
(2) Priewisch, B.; Rück-Braun, K. Efficient Preparation of Nitrosoarenes for the Synthesis of Azobenzenes. *J. Org. Chem.* **2005**, *70*, 2350-2352.
(3) Bléger, D.; Dokić, J.; Peters, M. V.; Grubert, L.; Saalfrank, P.; Hecht, S. Electronic Decoupling Approach to Quantitative Photoswitching in Linear Multiazobenzene Architectures. *J. Phys. Chem. B* **2011**, *115*, 9930-9940.
(4) Li, B.; Zhang, B.; Zhang, X.; Fan, X. Regio-selective synthesis of diversely substituted benzo[a]carbazoles through Rh(iii)-catalyzed annulation of 2-arylindoles with α-diazo carbonyl compounds. *Chem. Commun.* **2017**, *53*, 1297-1300.
(5) Deiana, M.; Pokladek, Z.; Dudek, M.; Mucha, S. G.; Mazur, L. M.; Pawlik, K.; Mlynarz, P.; Samoc, M.; Matczyszyn, K. Remote-control of the enantiomeric supramolecular recognition mediated by chiral azobenzenes bound to human serum albumin. *Phys. Chem. Chem. Phys.* **2017**, *19*, 21272-21275.
